# Glass promotes the differentiation of neuronal and non-neuronal cell types in the *Drosophila* eye

**DOI:** 10.1371/journal.pgen.1007173

**Published:** 2018-01-11

**Authors:** Carolyn A. Morrison, Hao Chen, Tiffany Cook, Stuart Brown, Jessica E. Treisman

**Affiliations:** 1 Skirball Institute for Biomolecular Medicine and Department of Cell Biology, NYU School of Medicine, New York, NY, United States of America; 2 Department of Cell Biology, NYU School of Medicine, New York, NY, United States of America; 3 Center of Molecular Medicine and Genomics and Department of Ophthalmology, Wayne State University School of Medicine, Detroit, MI, United States of America; National Centre for Biological Sciences, TIFR, INDIA

## Abstract

Transcriptional regulators can specify different cell types from a pool of equivalent progenitors by activating distinct developmental programs. The Glass transcription factor is expressed in all progenitors in the developing *Drosophila* eye, and is maintained in both neuronal and non-neuronal cell types. Glass is required for neuronal progenitors to differentiate as photoreceptors, but its role in non-neuronal cone and pigment cells is unknown. To determine whether Glass activity is limited to neuronal lineages, we compared the effects of misexpressing it in neuroblasts of the larval brain and in epithelial cells of the wing disc. Glass activated overlapping but distinct sets of genes in these neuronal and non-neuronal contexts, including markers of photoreceptors, cone cells and pigment cells. Coexpression of other transcription factors such as Pax2, Eyes absent, Lozenge and Escargot enabled Glass to induce additional genes characteristic of the non-neuronal cell types. Cell type-specific *glass* mutations generated in cone or pigment cells using somatic CRISPR revealed autonomous developmental defects, and expressing Glass specifically in these cells partially rescued *glass* mutant phenotypes. These results indicate that Glass is a determinant of organ identity that acts in both neuronal and non-neuronal cells to promote their differentiation into functional components of the eye.

## Author summary

Distinct cell types within an organ are specified by the activity of cell type-specific transcription factors, which activate distinct gene networks that confer the appropriate cellular identities. The *Drosophila* eye is derived from a field of equivalent progenitors that develop into photoreceptor neurons as well as non-neuronal cone and pigment cells. The zinc finger transcription factor Glass was thought to solely determine the photoreceptor identity. Here we show that Glass acts not only in photoreceptors, but also in the non-neuronal support cells of the eye, promoting the normal differentiation of each cell type. Loss of Glass in non-neuronal cells results in developmental defects, and these defects can be rescued by restoring Glass specifically to those cell types. We used misexpression assays to show that Glass is sufficient to induce markers of photoreceptors, cone cells and pigment cells, and we identified certain transcription factors that may cooperate with Glass to specify these non-neuronal cells. These results suggest that the role of Glass is to give each cell type its identity as a component of the eye. The transcriptional code that defines cell identities must thus include factors such as Glass that mark the cell as belonging to a specific organ.

## Introduction

Cell fate specification is achieved by integrating extrinsic signals with intrinsic transcriptional and epigenetic networks [1–3]. Certain transcription factors have been described as “master regulators” for their ability to specify entire organs [4–6], while downstream “terminal selectors” activate genes necessary to confer a specific cellular identity [7–9]. At intermediate levels of the hierarchy, other transcription factors endow progenitor cells with the ability to give rise to broad categories of cell types distinguished by their function or position [4, 10, 11]. In general, these mechanisms are thought to gradually narrow the fate choices available to each progenitor cell. It is not known whether distinct cell types within the same organ may share a common transcriptional signature.

The *Drosophila* eye consists of photoreceptor neurons and non-neuronal support cells that develop from a single field of uncommitted progenitor cells in the larval eye imaginal disc [12, 13], which is specified by a network of transcription factors encoded by the retinal determination genes [14]. Differentiation of the eye disc proceeds in a posterior to anterior wave driven by Hedgehog (Hh) signaling and led by an indentation known as the morphogenetic furrow [12, 15, 16]. This process gives rise to regularly spaced clusters of cells in which progenitor cells are sequentially specified as photoreceptors R1-R8 and the four lens-secreting cone cells [17, 18]. The remaining cells rearrange their positions and reduce their numbers during pupal development to produce a lattice of optically insulating pigment cells and mechanosensory bristles [19, 20]. Signaling between these differentiating cells, most notably through the Epidermal Growth Factor Receptor (EGFR) and Notch pathways, plays an important role in assigning uncommitted cells to photoreceptor, cone or pigment cell fates [21]. Each cell’s transcription factor content is also critical in determining how it responds to these signals [22–24].

The zinc finger transcription factor Glass (Gl) has been assigned a central role in specifying photoreceptor identity downstream of the retinal determination gene network [25–27]. In *gl* mutants, presumptive photoreceptors acquire neuronal characteristics, but fail to differentiate and do not express Rhodopsins or the phototransduction machinery [25, 27, 28]. These studies support the model that Gl drives the transition from a neuronal to a photoreceptor cell fate. Nevertheless, Gl expression is not confined to photoreceptors; it is present in all cells in the eye disc posterior to the morphogenetic furrow, and is dynamically expressed in all cell types during pupal development [26, 29]. While cone cells and pigment cells are still present in *gl* mutants, their numbers and morphology are abnormal, giving rise to the “glassy” phenotype that was the basis for the original identification of *gl*, and pigmentation is reduced [25, 30]. These defects have been considered to be a secondary consequence of abnormal photoreceptor development, as early studies suggested that Gl function was negatively regulated in non-neuronal cells [29]; however, its possible autonomous role in the development of these cell types has not been addressed.

Misexpression of Gl in the larval central nervous system has been shown to induce the expression of a subset of photoreceptor-specific genes [27]. As a first step towards investigating whether Gl function is restricted to neuronal lineages, we compared the effects of misexpressing it in different developmental contexts. We found that Gl was able to induce expression of the photoreceptor-specific gene *chaoptin* (*chp)* both in neuroblasts in the brain and in non-neuronal epithelial cells of other imaginal discs, independently of the retinal determination gene network. Transcriptomic analysis revealed distinct but overlapping sets of genes induced in these two cellular contexts, including genes characteristic of non-neuronal cone and pigment cells. Gain-of-function, rescue and loss-of-function experiments all supported autonomous roles for Gl in the normal differentiation of these non-neuronal cells of the eye as well as of photoreceptors. Furthermore, we identified several elements of a transcription factor network that cooperates with Gl to induce genes characteristic of cone and pigment cells. These results indicate that Gl is not a photoreceptor-specific factor, but a determinant of organ identity that is reiteratively used to promote the terminal differentiation of multiple cell types in the eye.

## Results

### Gl is sufficient to induce ectopic Chp expression in both neuronal and non-neuronal cells

**Fig 1:**
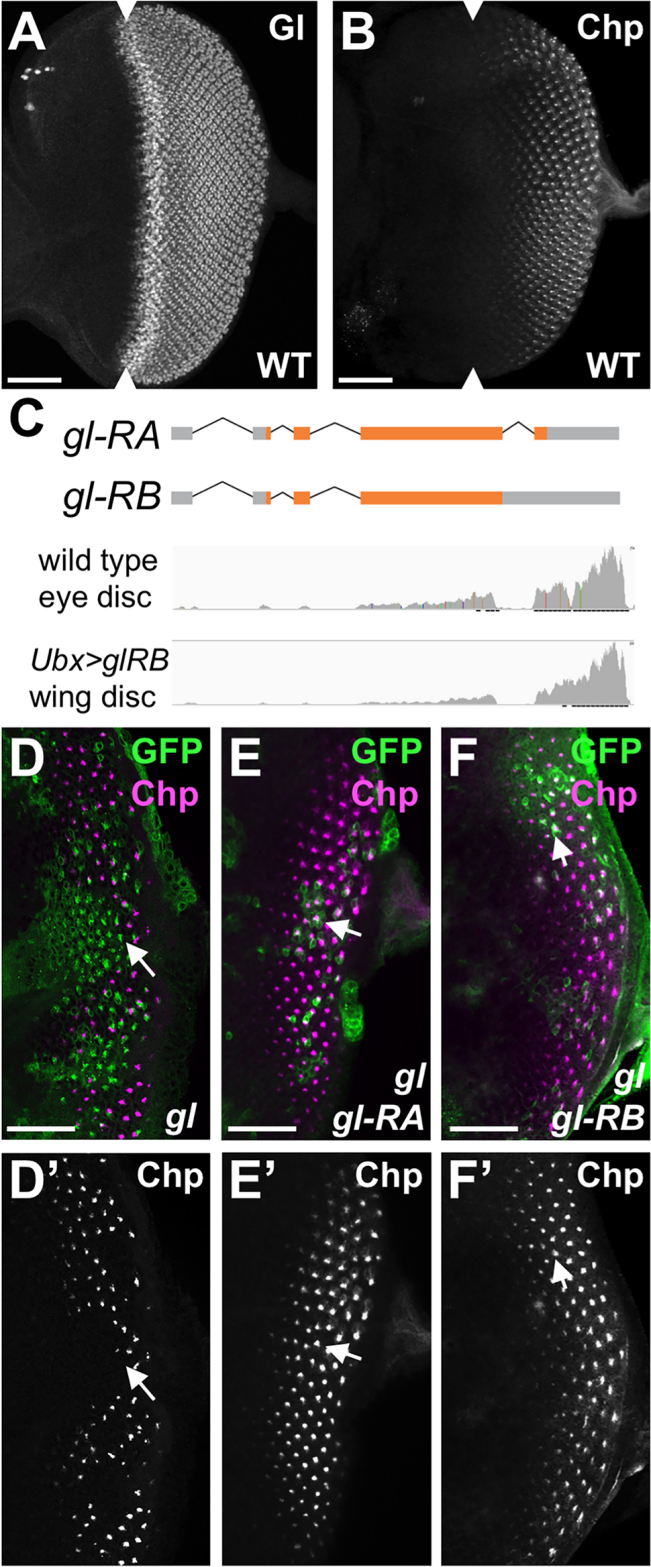
*gl* produces a single functional transcript. (A, B, D-F) show third instar larval eye imaginal discs stained with anti-Gl (A) and anti-Chp (B, D’-F’, magenta in D-F). Anterior is to the left in this and all subsequent figures. (A, B) wild type eye discs. Gl is expressed earlier than Chp. Arrowheads mark the morphogenetic furrow. (C) shows a diagram of the transcripts annotated on Flybase. *gl-RB* is predicted to retain an intron. Reads from RNA-Seq analysis of wild type eye discs and wing discs expressing UAS-*gl-RB* in clones of cells are aligned to the diagram and show no reads corresponding to this intron, indicating that it is spliced out in vivo (D-F) *gl*^*60j*^ homozygous mutant clones are marked by expression of GFP (green) and also express *UAS-gl-RA* (E) or *UAS-gl-RB* (F). Chp expression is absent from *gl* mutant clones (D) but restored by either transgene (E, F). Arrows indicate representative clones. Scale bars: 30μm in D-F; 50μm in A,B.

Gl is expressed in differentiating cells in the eye imaginal disc (Fig 1A) and is required for the expression of photoreceptor-specific genes such as *chaoptin* (*chp*) (Fig 1B and 1D) in cells that have been specified to become neurons [25]. However, Gl expression has also been noted in the non-neuronal cone cells and pigment cells of the eye [26, 29]. To test whether Gl activity is limited to neuronal cells, we generated two transgenic lines, UAS-*gl-RA* and UAS-*gl-RB*, in which the UAS promoter drives each of the *gl* isoforms annotated in Flybase (Fig 1C). *gl-RA* encodes a protein of 604 amino acids, containing five zinc fingers of which only the final three are essential for DNA binding [26, 31], and could also produce a 679 amino acid protein, Gl-RC, by readthrough of the stop codon. *gl-RB* is predicted to retain an intron that would introduce a frameshift leading to protein termination in the middle of the last zinc finger. However, our RNA-seq data indicated that the intron in *gl-RB* was spliced out *in vivo* (Fig 1C), suggesting that its apparent retention was an artifact of cDNA preparation. Expressing either Gl isoform in *gl* mutant clones restored Chp expression, confirming that both transgenes are functional (Fig 1E and 1F). We used the two transgenes interchangeably in subsequent experiments.

**Fig 2:**
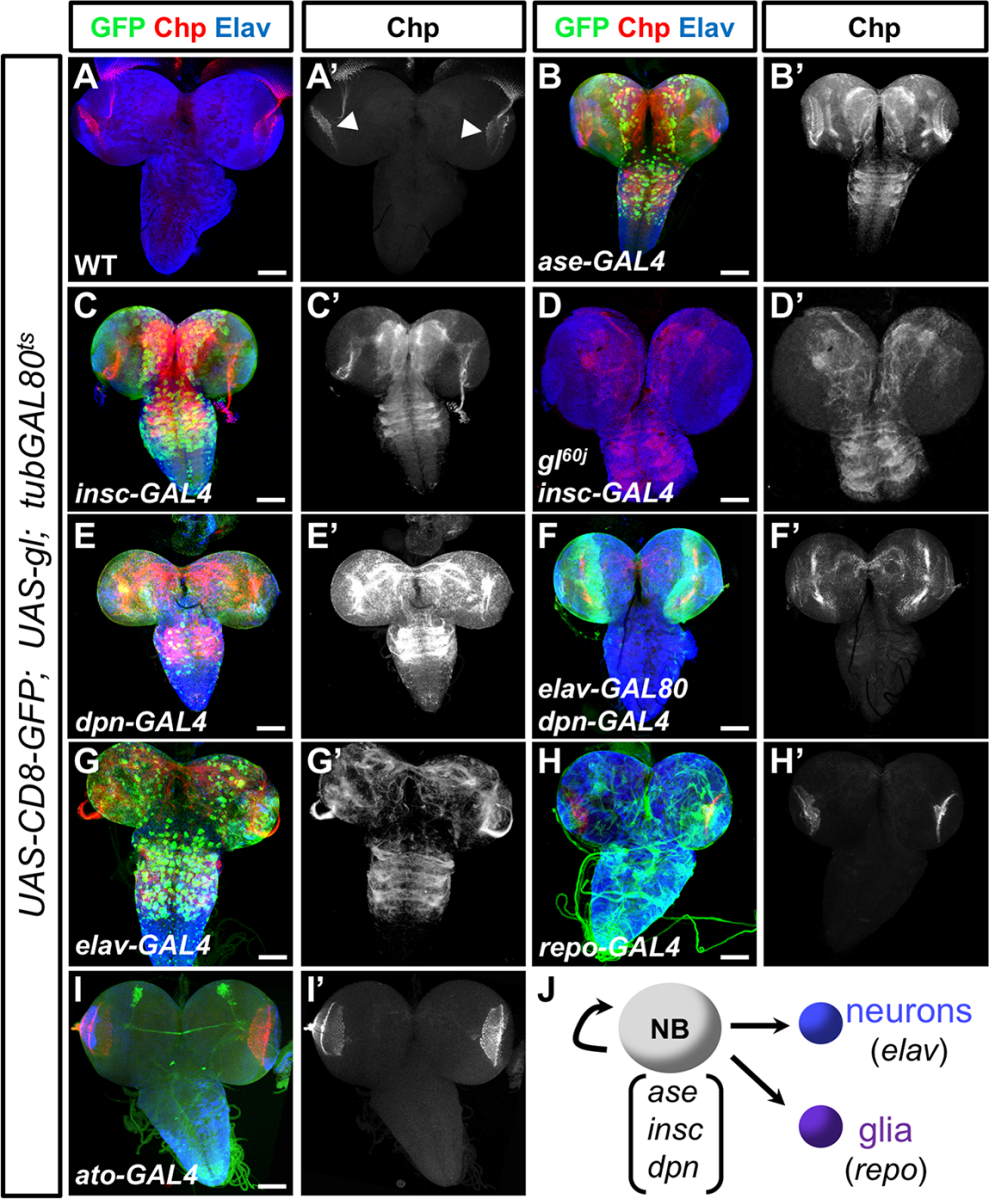
Gl can induce Chp when expressed in neuroblasts and neurons. (A-I) show third instar larval central nervous systems stained with anti-Chp (A’-I’, red in A-I) and anti-Elav (blue). The patterns of GAL4 drivers are shown by coexpressed *UAS-mCD8GFP* (green). (A) in a wild type CNS, Chp staining is restricted to photoreceptor axons innervating the optic lobes (arrowheads). (B-I) *UAS-gl* is driven by *ase-GAL4* (B), *insc-GAL4* (C), *insc-GAL4* in a *gl*^*60j*^ mutant background (D), *dpn-GAL4* (E), *dpn-GAL4* with *elav-GAL80* (F), *elav-GAL4* (G), *repo-GAL4* (H), or *ato-GAL4* (I). *tub-GAL80*^*ts*^ was used to inhibit GAL4 activity during early development; for *insc-GAL4* and *dpn-GAL4*, larvae were shifted to 29°C to inactivate GAL80 after 3 days at 18°C. *ase-GAL4* larvae were raised continuously at 29°C and *repo-GAL4* larvae were shifted to 29°C after 4 days at 25°C. (J) is a schematic indicating which GAL4 lines are expressed in neuroblasts (NB) or their neuronal or glial progeny. All three neuroblast drivers induce Chp in the central nervous system (B, C, E), but this induction is restricted by turning off Gl expression in differentiated neurons with *elav-GAL80* (F). Gl can induce Chp when expressed in neurons (G) but not glia (H) or sensory organ precursors (I). (D) shows the condition used for RNA-seq analysis; endogenous Chp is absent from the photoreceptor axons. Scale bars: 100μm.

As Gl was thought to drive the transition from a neuronal to a photoreceptor cell fate, we first tested the effects of misexpressing it in neural progenitors in the developing embryo and the larval brain. Endogenous Chp expression in the embryo is confined to larval photoreceptors in the Bolwig organ (S1A Fig), and the protein is present in photoreceptor axons and their terminals in the optic lobes of the third instar larval brain (Fig 2A). We found that misexpressing Gl in neuroblasts using *asense* (*ase*)-GAL4, *deadpan* (*dpn*)-GAL4 or *inscuteable* (*insc*)-GAL4 drivers, together with a temperature-sensitive form of GAL80 to bypass early lethality, led to induction of Chp in some cells in the central nervous system (CNS) of the developing embryo (S1B Fig) and in the central region of the larval brain and ventral nerve cord (Fig 2B, 2C and 2E).

The expression of Chp and Gl was not confined to cells that expressed UAS-CD8-GFP from the neuroblast driver (S2B–S2D Fig). As Gl can autoregulate its expression [26, 32], this observation suggested that Gl could be maintained in differentiating cells derived from the neuroblast lineage, and continue to induce ectopic Chp expression in these cells. To determine whether target gene induction in differentiating neuroblast progeny required sustained expression of Gl, we expressed Gl in neuroblasts but blocked its expression in differentiating neurons with GAL80 driven by the pan-neuronal *elav* promoter. Under these conditions, we no longer observed ectopic Chp expression in the ventral nerve cord (Fig 2F), indicating that Gl expression must be maintained to induce Chp in this region. Driving Gl expression specifically in differentiated neurons with *elav-*GAL4 also led to ectopic expression of Chp in the embryonic and larval CNS (Fig 2G, S1C Fig). In contrast, misexpressing Gl in glial cells with *reversed polarity* (*repo)*-GAL4 or in sensory organ precursors with *atonal* (*ato)*-GAL4 did not result in ectopic Chp expression (Fig 2H and 2I, S1D and S1E Fig). These results indicate that Gl is not sufficient for *chp* transcription, but acts in combination with other sequence-specific transcription factors or chromatin regulators that are present in a subset of neuronal cell types.

**Fig 3:**
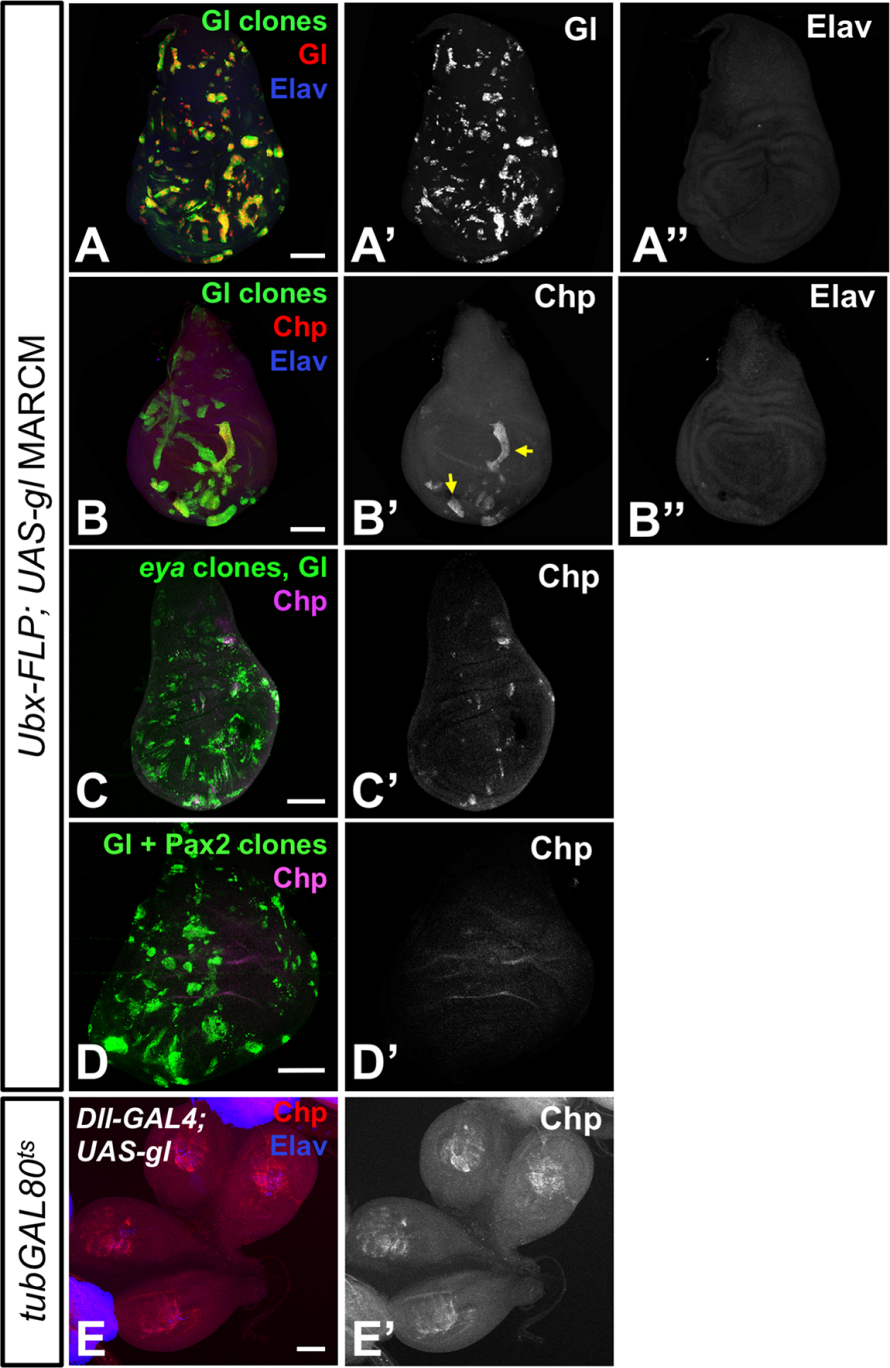
Gl can induce Chp when expressed in epithelial cells. (A-D) third instar wing imaginal discs in which *UAS-gl* is expressed with *tubulin (tub)-GAL4* in clones of cells generated using *Ubx-FLP* and marked by coexpression of *UAS-mCD8GFP* (green). (C) *UAS-gl* is expressed in *eya*^*E18B*^ mutant clones; (D) *UAS-gl* is coexpressed with *UAS-Pax2*. (E) shows leg discs with Gl misexpression in the central region driven by *Dll-GAL4*, with *tub-GAL80*^*ts*^ used to inhibit GAL4 activity for the first 3 days of development. Discs are stained with anti-Gl (A’, red in A), anti-Chp (B’, C’, D’, red in B, magenta in C, D) and anti-Elav (A”, B”, blue in A, B, E). Gl induces Chp, but not Elav, in wing and leg discs. Arrows in (B’) indicate some regions of Chp induction. Scale bars: 100μm.

A previous study suggested that Gl function was negatively regulated in non-neuronal cells, as ubiquitous Gl expression activated a reporter driven by Gl binding sites derived from the proximal enhancer of *Rhodopsin 1* (*Rh1*) specifically in neurons [29]. However, during normal eye development Gl is expressed earlier than neuronal markers such as Elav [26] (S4B Fig), and its expression is maintained in non-neuronal cone and pigment cells through early pupal stages [26, 29]. We therefore tested the effect of misexpressing Gl in developing leg and wing imaginal disc cells, which are epithelial progenitors similar to the first cells that express Gl in the eye disc. When we used *Ultrabithorax* (*Ubx*)-FLP and the MARCM system to express Gl in clones of epithelial cells in the wing imaginal disc (Fig 3A), we detected Chp expression within the clones (Fig 3B). Although not all of the clones misexpressing Gl activated Chp, we did not observe any consistent positional bias; the variability may have been related to the timing of Gl induction, as *Ubx*-FLP is active throughout wing disc development [33]. The cells that expressed Gl and Chp did not misexpress Elav (Fig 3A and 3B), indicating that they were neither originally fated to become neurons, nor converted to a neuronal fate by Gl. Similarly, when we misexpressed Gl in the leg disc using the *Distal-less* (*Dll*) driver, we observed ectopic Chp expression in Elav-negative cells (Fig 3E). Gl is thus capable of activating the transcription of photoreceptor-specific genes in non-neuronal epithelial cells.

During normal eye development, *gl* expression is under the direct transcriptional control of Sine oculis (So), a retinal determination factor that forms a compound transcription factor with Eyes absent (Eya) [27, 34]; *gl* can also be induced by the combination of Eya and Dachshund [35]. Consistent with the absence of Eya expression in the wild type wing disc epithelium [36], we found that Gl was still able to induce Chp when expressed in *eya* mutant clones (Fig 3C). This result is consistent with Gl acting as a downstream effector of the retinal determination gene network.

### Gl induces common and distinct sets of genes in neuronal and non-neuronal cells

To determine how a neuronal or non-neuronal cell context might influence Gl activity, we used RNA-Seq to examine changes in the transcriptome induced by Gl misexpression. For the neuronal misexpression condition, we used *gl* mutant third instar larval brains in which Gl expression was driven in neuroblasts by *insc*-GAL4 in combination with *tubulin (tub)*-GAL80^ts^ (Fig 2D). Using a *gl* mutant removed the contribution of clock cells in the brain that express Gl [37] and of mRNAs present in the photoreceptor axons that innervate the optic lobes. For epithelial misexpression, we used larval wing discs in which clones of cells generated with *Ubx*-FLP expressed Gl under the control of *tubulin-GAL4* (Fig 3A and 3B). For comparison, we also sequenced the transcriptomes of wild-type larval eye discs, wing discs, wild-type and *gl* mutant brains.

**Fig 4:**
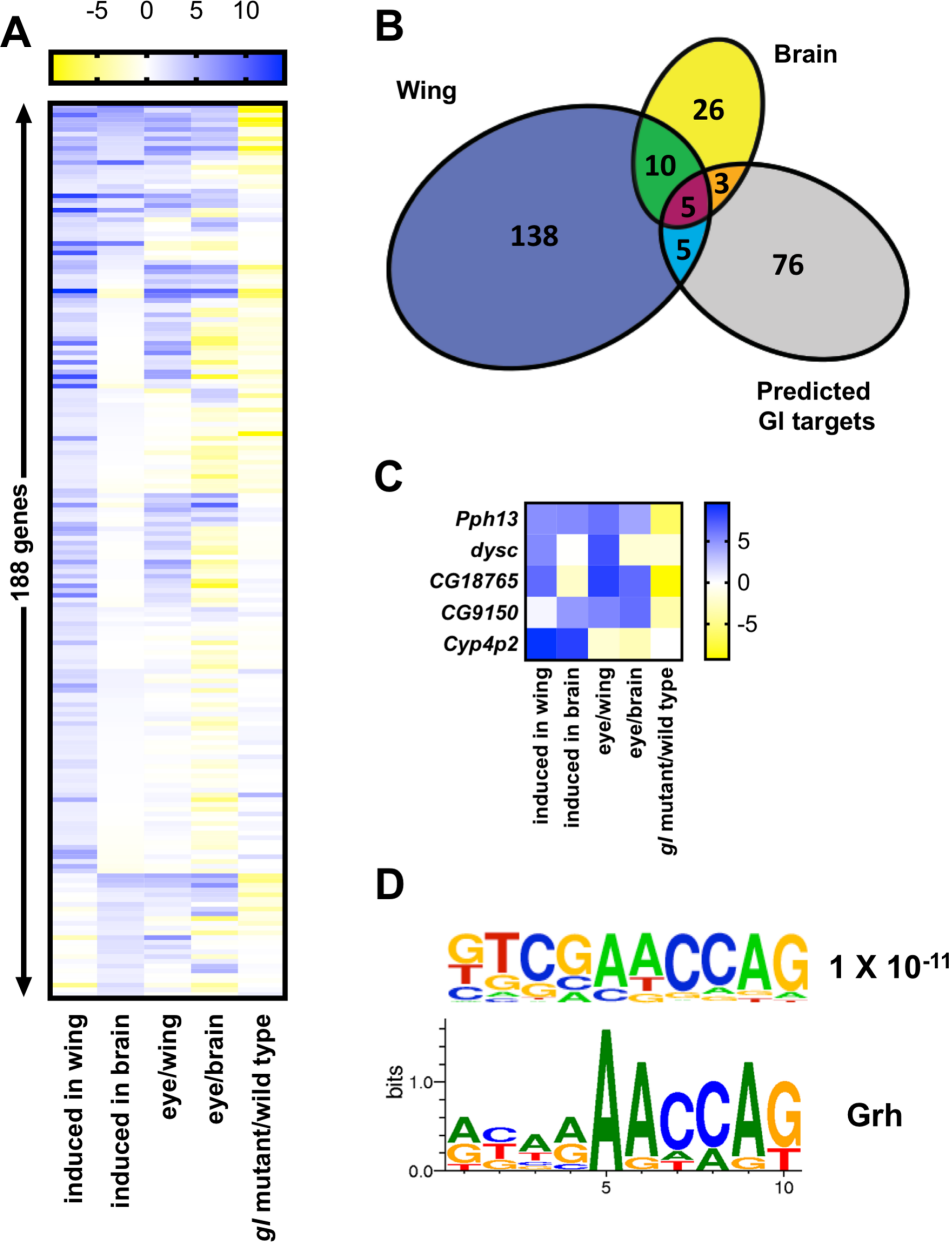
Gl induces more genes in the wing disc than the brain. **(**A) is a heat map of 188 significantly induced genes, indicating fold changes (log_2_) in *Ubx-FLP>gl* wing disc/wild type wing disc (induced in wing), *insc>gl* in *gl* brain/*gl* brain (induced in brain), wild type eye disc/wild type wing disc (eye/wing), wild type eye disc/wild type brain (eye/brain) and *gl* mutant eye disc/wild type eye disc (data from [38]). (B) Euler diagram drawn with APE showing the overlap of the 158 genes induced by Gl in the wing disc, the 44 genes induced by Gl in the brain, and the 89 Gl target genes predicted by [40], not including *gl* itself. The stringent cutoffs we used to classify genes as induced probably underestimate the actual overlap. (C) Heat map showing examples of different patterns of gene induction. *Pph13* is induced in both tissues, enriched in the eye disc compared to the wing disc and brain, and reduced in *gl* mutants, making it a core Gl target gene. *dyschronic* (*dysc)* is induced by Gl in the wing disc but not the brain, and its levels are high in the wild type brain compared to the eye disc. *CG18765* and *CG9150* are induced specifically in the wing and the brain, respectively, despite low basal levels in the other tissue. *Cyp4p2* is induced by ectopic Gl but is not enriched in the eye disc or dependent on endogenous *gl*. (D) A motif that was enriched in the regulatory regions of genes induced by Gl with p<10^−11^ (top) is a close match to the binding site for Grh taken from Fly Factor Survey (bottom).

Fig 4A shows a heat map of 188 genes that were induced at least two-fold (with p<0.01) in Gl-misexpressing wing discs and/or brains, with additional cutoffs to eliminate genes that were expressed at very low levels or had a large variance between the triplicate samples (S1 Table). A core set of Gl target genes were induced in both tissues, enriched in wild-type eye discs compared to wing discs or brains, and reduced in *gl* mutant eye discs [38]. These include genes involved in photoreceptor differentiation, phototransduction, neurotransmitter reception, and pigment synthesis. In addition, we detected induction of genes with no previously described function in eye development (Table 1, S1 Table), but that are highly enriched in photoreceptors or cone cells in the developing or adult *Drosophila* eye [39].

**Table 1:** Genes induced by Gl either broadly or tissue-specifically.

Gene	Induced in wing	Induced in brain	Enriched in eye/wing	Enriched in eye/brain	Reduced in *gl* mutant eye	Function
**Core target genes**						
Pph13*	5.1	5.3	6.3	4.2	-9.3	Homeodomain transcription factor, regulates photoreceptor-specific genes
inaC*	6.5	2.2	7.8	6.9	-8.7	Protein kinase C involved in phototransduction
HisCl1*	4.3	2.1	6.2	2.3	-4.7	Histamine receptor
Ekar*	5.0	2.4	6.2	4.2	-6.4	Glutamate receptor
*cd*†	5.1	4.2	4.7	2.4	-8.3	Haem peroxidase, ommochrome synthesis
*CG5653*†	5.3	8.6	7.5	7.1	-8.8	Polyamine oxidase
*Iris*	6.2	6.6	2.1	3.8	-9.3	Retroviral envelope fusion protein
*CG30101*†	1.8	3.3	2.3	3.6	-1.1	Cell wall protein
*CG17264*	4.0	2.4	2.6	2.9	-2.3	CUB domain protein
Culd*	0.7	1.7	1.7	2.8	-1.7	CUB-LDL protein, promotes Rhodopsin and TrpL endocytosis
*CG3777*	1.2	1.9	1.9	3.8	-0.15	Mucin/flocculation protein
**Specifically induced in the wing**						
Cpr51A*	2.2	0.1	4.1	3.4	-0.6	Cuticle protein
*sls*†	2.2	-0.3	2.	1.6	-1.5	Titin/Kettin
*Cyp4e1*	3.0	0.2	1.5	1.0	-2.2	Cytochrome P450
*blanks*	4.9	-0.3	6.2	0.8	-3.7	RNA-binding protein
*CG18765*	6.7	-2.4	8.5	6.7	-6.4	Protein kinase
CG13071*	13.9	-1.9	9.7	9.7	-7	Unknown
**Specifically induced in the brain**						
*Dot*	-0.2	1.0	1.1	2.2	-1.4	Ecdysone synthesis
*obst-B*†	-0.3	1.6	0.9	5.6	-1.1	Chitin binding
*CG13360*	-1.1	2.0	0.8	2.9	-3.8	Protein kinase

Numbers are log_2_ fold changes in conditions labeled as in Fig 4. Core target genes are induced in both tissues and enriched in eye discs compared to wing discs or brain with p<0.02, and reduced in *gl* mutant eye discs. Wing-specific genes are induced in the wing disc and unchanged or reduced in the brain, enriched in eye discs compared to wing discs, and reduced in *gl* mutant eye discs. Brain-specific genes are induced in the brain and unchanged or reduced in the wing disc, enriched in eye discs compared to brains, and reduced in *gl* mutant eye discs.

*Genes among the top 2000 most enriched in adult photoreceptors relative to cone cells

†genes among the top 2000 most enriched in adult cone cells relative to photoreceptors [39].

Surprisingly, Gl induced many more genes in the wing disc than in the brain (Fig 4A and 4B). Some of the genes induced only in the wing disc are already as highly expressed in wild-type brains as in eye discs (Fig 4C). However, other genes were induced in only one of the two tissues even though endogenous expression was low in both (Fig 4C; Table 1), presumably reflecting regulation by tissue-specific activators or repressors. Two previous studies predicted a set of genes likely to be direct targets of Gl activation based on the presence of clustered Gl binding sites in their regulatory regions and either reduced expression in *gl* mutant eye discs, or covariation with Gl across a variety of cell types and genetic conditions [38, 40]. Although some of these predicted target genes were induced by Gl misexpression in one or both tissues, many were not. We found only 13 of the 89 Gl targets predicted by [40] were significantly induced in either condition (Fig 4B, S3 Fig, S1 Table). One reason for this could be that the region searched for Gl binding sites included the first intron [38], which varies dramatically in length across different genes. The presence of potential Gl binding motifs is more likely to be significant in short than long sequences, and among the predicted target genes the length of the first intron was inversely correlated with the probability that the gene was induced in our experiments (S3A and S3B Fig). We also note that this prediction method missed the validated direct target genes *PvuII-PstI homology 13* (*Pph13*) and *prospero (pros)* [27, 28, 30].

The ability of Gl to induce genes that contain Gl binding motifs is also likely to depend on their responsiveness to other transcriptional activators and repressors present in the cell. A search for motifs that were enriched in the regulatory regions of genes induced by Gl, relative to predicted targets that were not induced, identified a motif matching the binding site for Grainyhead (Grh) (Fig 4D), a transcription factor that correlates with sites of open chromatin in the eye disc [40]. This suggests that chromatin structure controls the access of Gl to its binding sites.

### Gl acts autonomously in non-neuronal cells in the eye disc

**Fig 5:**
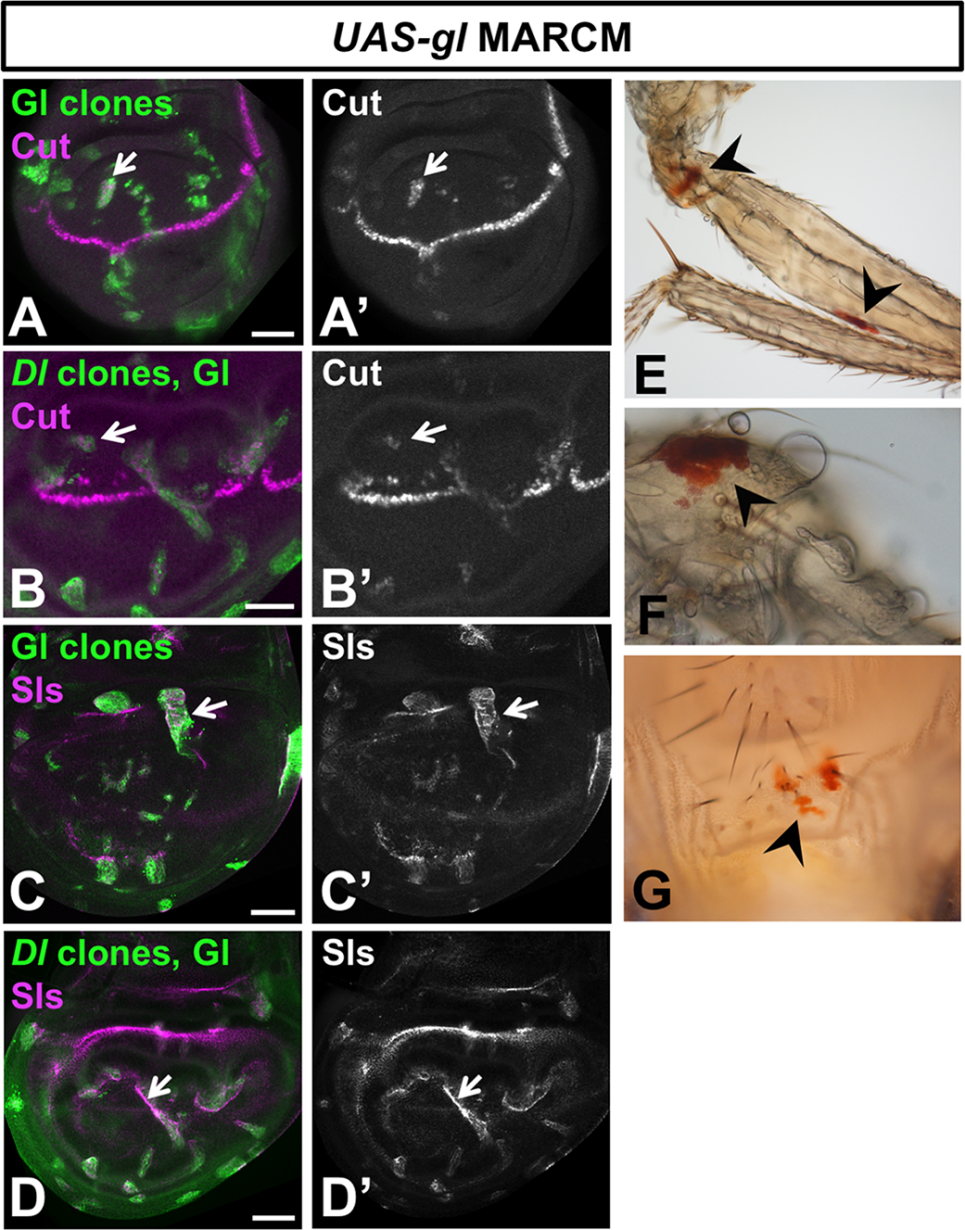
Gl induces cone and pigment cell markers. (A-D) wing discs in which *UAS-gl* is expressed in wild type (A, C) or *Dl*^*RevF10*^ (B, D) clones induced by *Ubx-FLP*, marked with GFP (green) and stained with anti-Cut (A’, B’, magenta in A, B) or anti-Sls (C’, D’, magenta in C, D). The cone cell markers Cut and Sls are induced in Gl-expressing clones (arrows) even if these are unable to activate the Notch pathway in neighboring cells. Adult legs (E, F) and abdomen (G) from flies in which Gl-expressing clones were induced with *ey-FLP* (E, F) or *Ubx-FLP* (G). Ectopic red pigment resembling eye pigment can be seen on the cuticle (arrows). Scale bars: 50μm.

**Fig 6:**
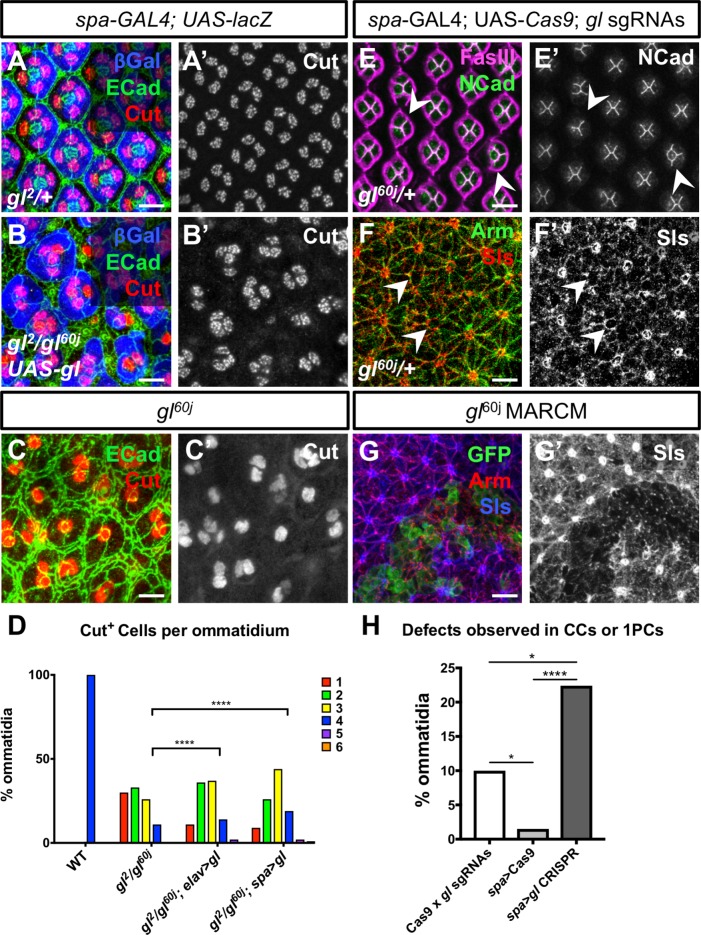
Gl can act autonomously in cone cells. (A-C) 42h APF pupal retinas stained with anti-Ecad (green), anti-Cut (A’-C’, red in A-C) and anti-β-galactosidase reflecting *UAS-lacZ* driven by *spa-GAL4* (blue in A, B). (A) *spa-GAL4; UAS-gl; gl/+*. (B) *spa-GAL4; UAS-gl; gl*^*2*^*/gl*^*60j*^. (C) *gl*^*60j*^. Expressing Gl in cone cells in a *gl* mutant background increases the number of cone cells per ommatidium and makes their arrangement more regular. (D) quantification of cone cell numbers in wild type, *gl* mutant and *gl* mutant rescued with *elav-GAL4*, *UAS-gl* or *spa-GAL4*, *UAS-gl*. n indicates number of ommatidia: wild type n = 82, *gl*^*2*^*/gl*^*60j*^ n = 79, *elav>gl* n = 83, *spa>gl* n = 226. ****p<0.0001, chi-squared test. (E, F) 42h APF *gl/+* retinas expressing *gl sgRNAs* and *UAS-Cas9* driven by *spa-GAL4* and stained with anti-FasIII (magenta in E) and anti-Ncad to mark cone cell contacts (E’, green in E) or anti-Arm to mark cell outlines (green in F) and anti-Sls (F’, red in F). Mutating *gl* in cone cells can cause defects in their patterning (E) and loss of Sls in cone cell feet (F, arrows). Some staining is still visible in the photoreceptor axons that surround the cone cell feet. (G) 42 h APF retinas with GFP-labeled *gl*^*60j*^ homozygous mutant clones. Retinas are stained with anti-GFP (green), anti-Arm (red) and anti-Sls (G’, blue in G). Sls is strongly expressed in cone cell feet and is lost in *gl*^*60j*^ clones. (H) quantification of patterning defects due to loss of Gl in cone cells and primary pigment cells (n = 143), compared to controls in which *UAS-Cas9* and *gl* sgRNAs are present without a GAL4 driver (n = 295) or *Cas9* is expressed in the absence of *gl* sgRNAs (n = 198). ****p<0.0001, *p<0.05, Fisher’s exact test. Scale bars: 10μm.

Our observation that Gl could activate gene expression in non-neuronal cells raised the possibility that its expression in the non-neuronal cell types in the eye [26, 29] might correlate with a functional role there. Further evidence suggesting a function for Gl in cone and pigment cells came from the nature of the genes that were induced by Gl misexpression. While many of these genes are known to act in photoreceptors, including *Pph13*, *Eye-enriched kainate receptor* (*Ekar*) and the phototransduction component *inactivation no afterpotential C* (*inaC*) [41–43], Gl also induced genes that are highly enriched in cone cells, such as *sallimus* (*sls*), *obstructor-B* (*obst-*B), *peste* (*pes*) and the glial marker *wrapper* [39, 44]. We confirmed that Gl-expressing clones in the wing disc autonomously induced Cut, a transcription factor specific to cone cells and bristle cells in the retina [45] (Fig 5A), and Sls (Fig 5C), which is enriched in cone cell feet in the pupal retina and lost from them in *gl* mutant clones (Fig 6G). Expression of these markers was not due to secondary induction of cone cells by Gl-expressing photoreceptors through Notch signaling [46], as it did not require the function of the Notch ligand Delta (Dl) in the Gl-expressing cells (Fig 5B and 5D). In addition to inducing cone cell markers, Gl misexpression led to the induction of pigment synthesis genes such as *cardinal* (*cd*) and *rosy* (*ry*) [47, 48]. Consistent with induction of these genes, misexpression of Gl in clones of cells with *eyeless* (*ey*)-FLP or *Ubx*-FLP led to the appearance of ectopic red pteridine pigment, normally only produced by pigment cells in the eye [19], on the adult legs and abdomen (Fig 5E–5G).

**Fig 7:**
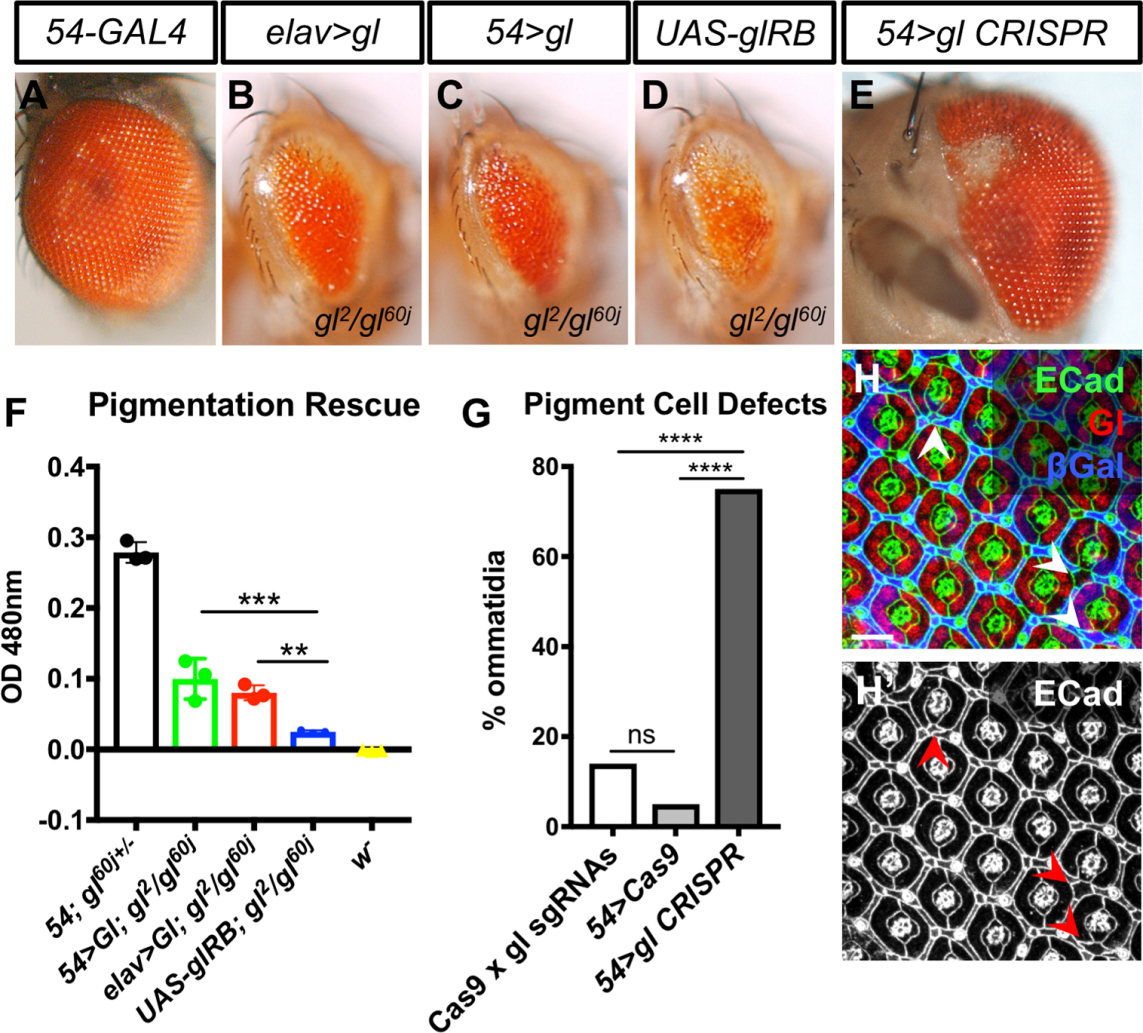
Gl can act autonomously in pigment cells. (A-E) show adult eyes; (A) *54-GAL4*, *UAS-lacZ*; (B) *elav-GAL4/UAS-gl*, *UAS-lacZ; gl*^*2*^*/gl*^*60j*^; (C) *54-GAL4/UAS-gl*, *UAS-lacZ; gl*^*2*^*/gl*^*60j*^; (D) *54-GAL4*, *UAS-lacZ/+*; *gl*^*2*^*/gl*^*60j*^; (E) *54-GAL4/gl sgRNAs; UAS-Cas9P2*, *gl*^*60j*^*/+*. Expressing Gl specifically in neurons or in the precursors of secondary and tertiary pigment cells in a *gl* mutant background increases the production of eye pigment, while mutating *gl* in pigment cells causes loss of pigment. (F) shows a quantification of pteridine pigment in the heads of *54-GAL4*, *UAS-lacZ/+; gl*^*60j*^*/+* controls; *54-GAL4*, *UAS-lacZ/UAS-gl*; *gl*^*2*^*/gl*^*60j*^ or *elav-GAL4*, *UAS-lacZ/UAS-gl*; *gl*^*2*^*/gl*^*60j*^ rescued flies; *UAS-gl*, *UAS-lacZ/+; gl*^*2*^*/gl*^*60j*^ *gl* mutants; and *w*^*1118*^ flies. **p<0.005, ***p<0.0001, unpaired two tailed t-test. (G) shows a quantification of ommatidial patterning defects when *UAS-Cas9* and *gl* sgRNAs are present with no GAL4 driver (n = 295) or when *Cas9* is expressed in pigment cells in the presence (n = 154) or absence (n = 226) of *gl sgRNAs*. ****p<0.0001, ns, not significant, Fisher’s exact test. (H) 42h APF *54-GAL4*, *UAS-Cas9/gl sgRNAs; gl*^*60j*^*/+* pupal retina stained with anti-Ecad (H’, green in H), anti-Gl (red in H), and anti-β-galactosidase reflecting *UAS-lacZ* driven by *54-GAL4* (blue in H). Examples of defects quantified in (G) are shown (arrows, H’). Scale bar: 10μm.

*gl* mutants exhibit numerous defects in cone and pigment cell differentiation. The number of cone cells is reduced (Fig 6C and 6D), there is significantly less eye pigmentation (Fig 7D and 7F) and the morphology of cone and pigment cells is abnormal [25, 28] (Fig 6C), giving the eye a glassy appearance. However, these defects have been thought to arise as a secondary consequence of abnormal photoreceptor differentiation, and a possible autonomous requirement for Gl in these cells has not been examined. To test whether endogenous Gl has an autonomous function in cone and pigment cells, we used our UAS-*gl* transgene to restore Gl to specific cell types in a *gl* mutant background. Expressing *gl* in cone cells and primary pigment cells with *sparkling* (*spa*)-GAL4 [49] or in interommatidial secondary and tertiary pigment cells with *54*-GAL4 [50] each produced a partial rescue of the mutant phenotype. Restoring Gl only in cone and primary pigment cells increased the number of cone cells per ommatidium (Fig 6B and 6D), consistent with an autonomous function of Gl within the cone cells that is independent of its role in photoreceptors. Restoring Gl only in the interommatidial pigment cells increased the level of pteridine eye pigments compared to *gl* mutant eyes with no rescue construct (Fig 7C, 7D and 7F). These results support an autonomous effect of Gl on the differentiation of the non-neuronal cell types of the eye. Restoring Gl specifically to photoreceptors with *elav*-GAL4 produced a similar partial rescue of cone cell number and pigmentation (Fig 6D, Fig 7B and 7F), indicating that Gl also acts upstream of the signals produced by photoreceptors that induce the differentiation of other cell types.

We next wished to remove *gl* function from specific cell types. As the *gl* transgenic RNAi lines available did not fully reproduce the *gl* phenotype even when expressed ubiquitously throughout eye development, we used the CRISPR-Cas9 method to generate somatic *gl* mutations in a cell-type specific manner [51]. We generated a transgenic line that expressed two synthetic guide RNAs (sgRNAs) targeting *gl* (S4A Fig), and crossed it to UAS-Cas9 in a heterozygous *gl* mutant background. Expression of Cas9 throughout the eye disc beginning early in development with *ey3*.*5*-FLP and *Act*>CD2>GAL4 [52] resulted in mosaic eyes with patches resembling the *gl* mutant phenotype (S4C and S4D Fig). A weaker phenotype was obtained when we removed Gl specifically from photoreceptors using *elav*-GAL4 (S4E–S4G Fig). A majority of Elav^+^ photoreceptors lost Gl expression, but Gl was still detected in the interommatidial cells, confirming that our CRISPR approach was cell type-specific (S4E and S4F Fig). Defects in the development of the non-neuronal cells were nevertheless observed (S2 Table), again consistent with reduced non-autonomous signaling by *gl* mutant photoreceptors.

To test the role of Gl in cone cells and primary pigment cells we used *spa*-GAL4 to express Cas9. Although Gl is not detectable in cone cells by 42h after puparium formation (APF) in the wild-type pupal retina, it is present during cone cell specification and in pupal primary pigment cells [29]. Gl expression was lost from a subset of presumptive cone cells marked by Pax2 in the third instar larval eye disc in *spa*-driven CRISPR mutagenesis (S4I Fig). At 42h APF we observed defects in the number and arrangement of cone cells and primary pigment cells, and perhaps as a secondary consequence, defects in the interommatidial cells (Fig 6E and 6H, S5A–S5E Fig, S2 Table). Although the expression of cone cell markers such as N-cadherin (Ncad) [53] and Fasciclin III (FasIII) [39] was unaffected (Fig 6E), Sls was depleted from cone cell feet when *gl* was mutated in these cells by driving Cas9 with *spa-GAL4* (Fig 6F). Expression of Cas9 with *54*-GAL4 resulted in loss of Gl from the interommatidial cells (S4K and S4L Fig), defects in the pigment cell lattice (Fig 7G and 7H, S5F–S5J Fig, S2 Table), and loss of pigment in patches in the adult eye (Fig 7E). These GAL4 lines were specific to the non-neuronal cells, as very few photoreceptor defects were observed (S5K Fig). These results demonstrate that Gl autonomously promotes the differentiation of all the cell types of the eye.

### Gl cooperates with other transcription factors to induce cell type-specific genes

**Fig 8:**
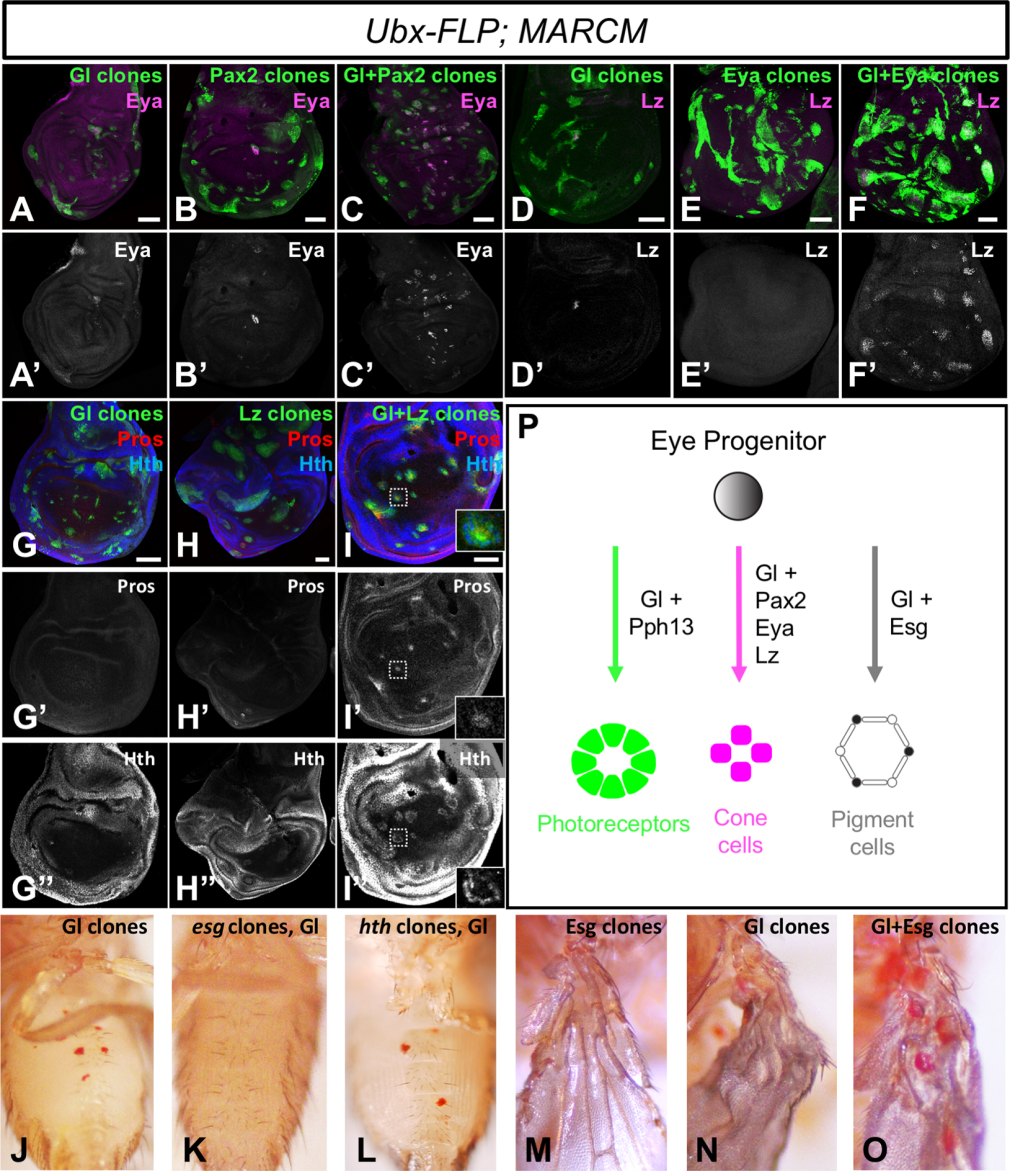
Gl cooperates with other transcription factors to induce cone and pigment cell genes. (A-I) show wing discs in which transcription factors are expressed in clones of cells generated with *Ubx*-FLP and positively labeled with GFP (green). (A, D, G) UAS-*glRB*; (B) UAS-*Pax2*; (C) UAS-*glRB* and UAS-*Pax2*; (E) UAS-*eya*; (F) UAS-*glRB* and UAS-*eya*; (H) UAS-*lz*; (I) UAS-*glRB* and UAS-*lz*. Discs are stained with anti-Eya (A’-C’, magenta in A-C), anti-Lz (D’-F’, magenta in D-F), anti-Pros (G’-I’, red in G-I) and anti-Hth (G”-I”, blue in G-I). Gl cooperates with Pax2 to induce Eya, with Eya to induce Lz, and with Lz to induce Pros and Hth. Inset in (I) shows an enlargement of the boxed region, showing cytoplasmic Pros surrounded by Hth-expressing cells. (J-M) adult flies with transcription factors misexpressed in clones generated with *Ubx*-FLP, showing ectopic red pigment on the abdomen (J-L) or wing (M-O). (J) UAS-*glRB*; (K) UAS-*glRB* in *esg*^*66B*^ mutant clones; (L) UAS-*glRB* in *hth*^*B2*^ mutant clones; (M) UAS-*esg*; (N) UAS-*glRB*; (O) UAS-*glRB* and UAS-*esg*. Esg, but not Hth, is necessary for Gl to induce pigment and enhances pigment induction by Gl. (P) model showing that Gl activates terminal differentiation genes in all the cell types of the eye, but cooperates with different transcription factors in each cell type.

Our finding that Gl acts autonomously in photoreceptors, cone cells and pigment cells raises the question of how it activates a different set of genes in each cell type. Gl has been shown to cooperate with the downstream transcription factor Pph13 to induce and maintain photoreceptor-specific gene expression [27, 32], and Orthodenticle also plays a role in regulating terminal differentiation of photoreceptors [41]. We therefore focused on identifying transcription factors that might help Gl to activate cone or pigment cell genes. Pax2 is a transcription factor that is specifically expressed in cone and primary pigment cells [54], independently of Gl (S4J Fig), and is required for their development [24, 54]. It also shares many predicted target genes with Gl [40]. We found that coexpressing Pax2 with Gl in clones in the wing disc prevented induction of the photoreceptor-specific gene *chp* (Fig 3D), consistent with the previously reported anti-neuronal function of Pax2 [24], and enabled the induction of *eya*, which was induced in very few cells by either factor alone (Fig 8A–8C). Eya is specifically expressed in non-neuronal cells at pupal stages [55] and has a late function in their terminal differentiation, independent of its early role in retinal determination [56, 57]. Consistent with this function, coexpressing Eya with Gl enabled it to induce *lozenge* (*lz*) (Fig 8D–8F), which encodes a transcription factor that is expressed in late-differentiating cells including the cone and pigment cells and is necessary for their differentiation [23, 58, 59]. Gl is necessary for *lz* expression [60] but not alone sufficient to induce it in the wing disc (Fig 8D).

Gl and Lz are known to cooperatively activate the expression of Pros, which cooperates with Pax2 to promote cone cell differentiation [24, 30], and are predicted to co-regulate many additional targets [40]. We found that in combination but not individually, Gl and Lz were able to induce ectopic *pros* and *homothorax* (*hth*) expression in the wing disc (Fig 8G–8I). Interestingly, Pros staining appeared largely cytoplasmic, a localization seen in mature cone cells but not in R7 photoreceptors, where it is nuclear [39, 61]. Hth is specific to pigment cells at pupal stages [62]. In clones misexpressing both Gl and Lz, Pros-expressing cells were often surrounded by Hth-expressing cells (Fig 8I), reflecting the relative positions of cone and pigment cells in normal development. Gl was still able to induce ectopic pigment when expressed in *hth* mutant clones (Fig 8L); however, Escargot (Esg), another transcription factor specific to pupal pigment cells [63] that is also predicted to coregulate Gl target genes [40], showed a clear cooperative interaction with Gl. Coexpressing Esg with Gl in clones generated with *Ubx-FLP* strongly enhanced ectopic pigment formation (Fig 8M–8O), while Gl failed to induce pigment when expressed in *esg* mutant clones (Fig 8K). Esg is generally thought to be a repressor [64], so its effect on the induction of pigment synthesis genes is likely to be indirect. Together, these results show that other transcription factors influence the set of target genes that Gl is able to activate, and suggest a preliminary model for the transcriptional control of cell type differentiation in the eye (Fig 8P).

## Discussion

Previous studies of Gl have described it as a determinant of photoreceptor cell fate [25, 27, 28]. Our data suggest that this characterization does not fully capture its function. We examined whether Gl was sufficient to drive uncommitted cells toward a differentiated photoreceptor cell identity. Although Gl could induce a subset of photoreceptor-specific genes, it did not initiate the entire photoreceptor differentiation program. Gl activated overlapping but distinct sets of genes in neuronal and non-neuronal cells, indicating that its function is not limited to neurons, but its effects are dependent on the cellular context. The results of loss-of-function, gain-of-function and rescue experiments show that in addition to its role in photoreceptors, Gl acts cell-autonomously to promote the normal differentiation of non-neuronal cone and pigment cells in the eye. Gl thus appears to have a parallel function in all the cell types of the eye, in combination with distinct transcription factors in each cell type, to give them their identities as components of an integrated organ (Fig 8P).

### Cell context influences the transcriptional activity of Gl

Context-dependent effects on transcription factor activity have been well documented [65–71]. Our transcriptomic analysis shows that Gl can activate some of its target genes in cells that have either a neuronal or an epithelial identity, while others are activated in only one of the two contexts. As Gl is expressed earlier than neuronal markers [26], it may act in epithelial progenitors in the eye disc prior to their neuronal differentiation. The developing eye and wing discs are distinguished by the expression of the retinal determination genes in the eye disc primordium [14]. One important function of this gene network is to induce Gl, which then directs eye disc cells to differentiate as components of the retina [27, 52]. The wing disc shares common signaling pathways with the eye disc, such as Hh, EGFR and Notch, which may enable Gl to activate some of its target genes in this context. Similarly, Decapentaplegic (Dpp) pathway activity appears to aid the ability of the “master transcriptional regulator” Eyeless/Pax6 to induce ectopic eye formation [35, 72].

The effect of Gl also depends on other cell type-specific transcription factors. A Gl binding site from the *Rh1* proximal enhancer drives more restricted reporter expression when adjacent sequences are included, supporting the existence of a repressor that can counteract activation by Gl [29]. The presence of such repressors or the absence of coactivators probably explains why only a few of the previously predicted Gl target genes [40] were induced by Gl misexpression in the wing disc or brain, and tissue-specific cofactors are likely to contribute to differential induction in the two contexts. We identified Pax2, Eya, Lz and Esg as transcription factors that can alter the spectrum of genes induced by Gl misexpression in the wing disc. In addition, the chromatin landscape may affect the availability of Gl binding sites. The enrichment of a Grh binding motif in genes that were induced by Gl suggests that Gl requires an open chromatin state to activate transcription [40], and is thus not a pioneer transcription factor [73]. Similarly, the *C*. *elegans* Gl homologue CHE-1, which is required for the expression of genes specific to the ASE gustatory neurons [74], can reprogram other cells into ASE neurons only when factors that promote chromatin-mediated repression are removed, indicating that its ability to activate target genes is influenced by their chromatin state [71, 75–77].

The duration of Gl expression may also influence its ability to activate target genes. Previous studies which concluded that Gl was not sufficient to activate photoreceptor-specific genes in non-neuronal cells used transient misexpression from a heat shock promoter [29]. However, we found that maintenance of Gl expression in the differentiated progeny of neuroblasts was necessary to induce ectopic *chp* expression. Extended expression may allow Gl to induce its target gene *Pph13*, which is necessary for the ectopic induction of genes such as *Rh1* and the phototransduction components *inactivation no afterpotential D* and *Arrestin 1* [27]. This feed-forward mechanism could contribute to the specificity of photoreceptor determination.

### Gl promotes the differentiation of multiple eye cell types

In general, cell fate specification is viewed as a series of decision points at which the expression of different transcription factors directs cells towards progressively restricted fates [10]. Downstream of factors that define broad spatial or temporal identities [78], “master regulators” such as Pax6 specify a field of multipotent progenitors within which patterned differentiation of all the cell types of the organ can occur [6]. Terminal selector genes are thought to be confined to specific cell types and directly induce their differentiated properties [8]. Gl was previously viewed as a terminal selector gene for photoreceptor identity [25, 27, 28]. However, Gl is present in cone and pigment cell precursors in addition to photoreceptors [26, 29] and regulates the expression of genes such as *lz* and *pros* that are expressed in both photoreceptors and cone cells [30, 60]. Our cell type-specific rescue and loss-of-function experiments show that the abnormal arrangement and differentiation of non-photoreceptor cells in *gl* mutant eyes is not simply a secondary consequence of the effect of *gl* on photoreceptors; instead, *gl* also acts autonomously in cone and pigment cells to regulate their number, arrangement and gene expression. In addition, ectopic Gl is capable of inducing genes specific to cone cells and the synthesis of red pteridine pigments that are normally made by secondary and tertiary pigment cells [19]. Gl thus appears to be a terminal differentiation factor for multiple cell types of the eye rather than a photoreceptor determinant. Few factors that confer organ identity on multiple distinct cell types have been described; for instance, differentiation of endocrine and exocrine cells in the pancreas appears to involve entirely distinct transcriptional regulatory networks [79]. In plants, however, different combinations of transcription factors produce different floral components, such that each transcription factor contributes to specifying multiple cell types of the flower [80].

As Gl itself does not distinguish photoreceptors from non-neuronal eye cells, its transcriptional targets must depend on other factors that control the identity of each cell type. The EGFR and Notch signaling pathways are important for recruiting both photoreceptor and non-photoreceptor cell types, and the transcription factors downstream of these pathways directly regulate cell type-specific genes [24, 81]. Although EGFR signaling has a direct or indirect effect on the differentiation of all cell types except R8 [17, 82, 83], the level of signaling can influence cell fate specification. For example, activation of the Sevenless receptor increases Ras-MAPK signaling in R7 relative to cone cells; this allows the repressor Tramtrack to be degraded, leading to high level expression of genes that promote neuronal identity [23, 84, 85]. Combinatorial effects of the two pathways contribute to cell type-specific expression of genes such as *Pax2* [59]. Our experiments suggest that Pax2 and Esg can modify the effects of Gl to promote the induction of cone and pigment cell genes respectively through a network of other downstream transcription factors.

The level of Gl could also affect which target genes it activates; Gl expression is reduced in cone cells during pupal development [29], and different levels of a transcription factor can specify different cell fates in other systems [86, 87]. In addition, the time of cell differentiation could play a role; for instance, neuroblasts express a temporal series of transcription factors that specify different identities in their progeny [88, 89]. Lz, a transcription factor that contributes to specifying R1, R6, R7 and the cone and pigment cells [22, 23, 58, 59], is a target of Gl regulation and is also predicted to act in combination with Gl to control many common target genes, suggesting that it functions as a feed-forward temporal determinant [40, 60, 90]. We found that Lz can indeed bias transcriptional activation by Gl towards genes expressed in later-differentiating cell types. The distinct effects of different combinations and levels of transcription factors and signaling pathways demonstrate how unique cell fates can be specified from a common pool of progenitors using few factors. Nevertheless, our understanding of cell type specification in the eye is probably still incomplete; for instance, analysis of the *spa* enhancer that drives *Pax2* expression in cone cells revealed many essential inputs in addition to the previously established regulation by EGFR signaling, Notch signaling and Lz [59, 91]. A fuller understanding of the network that modifies the transcriptional activity of Gl may hold the key to the problem of cell fate specification.

## Materials and methods

### *Drosophila* genetics

UAS-*gl-RB* was made by cloning the *gl* cDNA from clone GH20219 (*Drosophila* Genomics Resource Center) into pUASTattB using EcoRI and XhoI. A 1.5 kb NdeI/XhoI fragment lacking the intron was generated by overlap PCR and used to replace the corresponding region of UAS-*gl-RB* to generate UAS-*gl-RA*. Both constructs were integrated into the *VK37* attP site at position 22A3. The *gl* sgRNA sequences identified on www.flyrnai.org/crispr2/ [92], GCAGGATAGGCAGCCGACGC (*gl* gRNA 1) and TACCCACCGCTGCTGAGTCC (*gl* gRNA 2) were cloned into pCFD4 [51] by PCR and Gibson assembly, and the construct was integrated into the *attP40* site at 25C6. Injections and screening of transgenic flies were carried out by Genetivision.

Stocks used to generate clones were (1) UAS-*gl-RA*; *FRT82*, *gl*^*60j*^ (2) UAS-*gl-RB*; *FRT82*, *gl*^*60j*^ (3) UAS-*CD8-GFP*, *ey*-FLP; *tub*-GAL4, *FRT82*, *tub*-GAL80/TM6B (4) UAS-*CD8-GFP*, *Ubx*-FLP; *tub*-GAL4, *FRT82*, *tub*-GAL80/TM6B (5) UAS-*gl-RB*; *FRT82*, *P(w*^*+*^*)* (6) *FRT82*, *gl*^*60j*^ (7) UAS-*glRB*; *FRT82*, *Dl*^*RevF10*^ (8) *FRT42*; UAS-*Pax2* (9) *FRT42*, UAS-*glRB*; UAS-*Pax2* (10) UAS-*CD8-GFP*, *Ubx*-FLP; *FRT42*, *tub*-GAL80; *tub*-GAL4/TM6B (11) *FRT42*, UAS-*glRB* (12) *FRT42*, UAS-*glRB*; UAS-*eya* (13) *FRT42*; UAS-*eya* (14) UAS-*glRB*; *FRT82*, UAS-*lz* (15) *FRT82*, UAS-*lz* (16) *FRT42*, UAS-*glRB*; UAS-*esg* (17) UAS-*esg*; *FRT82* (18) UAS-*glRB*; *FRT82*, *hth*^*B2*^ (19) *esg*^*66B*^, *FRT40*, UAS-*glRB* (20) *eya*^*E18B*^, *FRT40*, UAS-*glRB* (21) UAS-*CD8-GFP*, *Ubx*-FLP; *FRT40*, *tub*-GAL80; *tub*-GAL4/TM6B. Stocks used for misexpression were (1) *dpn*-GAL4 (2) *elav*-GAL80; *dpn*-GAL4 (3) *elav*-GAL4 (4) *repo*-GAL4 (5) *ase*-GAL4 (6) *ato*-GAL4 (7) *insc*-GAL4 (8) UAS-*CD8-GFP*; UAS-*gl-RB*; *tub*-GAL80^ts^ (9) *Dll*-GAL4 (10) *insc*-GAL4; *tub*-GAL80^ts^, *gl*^*60j*^. Stocks used for rescue were (1) *spa*-GAL4; *gl*^*60j*^ (2) *54*-GAL4, UAS-*lacZ*; *gl*^*60j*^ (3) UAS-*gl-RB*, UAS-*lacZ*; *gl*^*2*^. Stocks used for CRISPR were (1) *ey3*.*5*-FLP, *Act*>CD2>GAL4; UAS-*Cas9P2*, *gl*^*60j*^/TM6B (2) *elav*-GAL4; UAS-*Cas9P2*, *gl*^*60j*^/SM6-TM6B (3) *spa*-GAL4; UAS-*Cas9P2*, *gl*^*60j*^/TM6B (4) *54*-GAL4, UAS-*lacZ*; UAS-*Cas9P2*, *gl*^*60j*^/SM6-TM6B (5) *gl sgRNAs* (*attP40*). Transgenic lines other than UAS-*gl* and *gl sgRNAs* are described in Flybase.

### Immunohistochemistry

Embryos and larval eye discs, wing discs and brains were stained as described [93, 94], fixing 30 min in 4% formaldehyde in 0.1M PIPES pH 7.0/2mM MgSO_4_/1mM EGTA for most antibodies but 45 min in 2% formaldehyde in 75mM lysine/370mM sodium phosphate pH 7.2/10mM NaIO_4_ for anti-Gl. Pupal retinas were fixed and stained as described [95]. Antibodies used were mouse anti-Gl (1:10; Developmental Studies Hybridoma Bank (DSHB), mouse anti-Chp (1:50; DSHB), chicken anti-GFP (1:300; Aves), rat anti-Elav (1:100; DSHB), mouse anti-Cut (1:10; DSHB), rabbit anti-β-galactosidase (1:5000; Cappel), rat anti-Ecad (1:10; DSHB), mouse anti-FasIII (1:10; DSHB), rat anti-Ncad (1:10; DSHB), rat anti-Kettin/Sls (1:200; Abcam), mouse anti-Eya (1:10; DSHB), mouse anti-Lz (1:10; DSHB), mouse anti-Pros (1:10; DSHB), rabbit anti-Hth (1:200) [96], rat anti-Pax2 (1:500) [24] and mouse anti-Arm (1:10; DSHB). Secondary antibodies were from Jackson Immunoresearch (Cy3 or Cy5 conjugates used at 1:200) or Invitrogen (Alexa488 conjugates used at 1:1000). Images were captured on a Leica SP5 confocal microscope and processed using ImageJ and Adobe Photoshop. Cone cell counts were performed using ImageJ.

### Pigment quantification

Pigment quantification was performed as described [97] with the following modification: for each sample, 30 heads from 3–5 day old adult females were homogenized in 0.5mL of 0.1M HCl in ethanol. OD_480_ values were obtained using the homogenizing solution as a blank.

### RNA-Seq

Larval tissue was isolated from 30 animals of each genotype in triplicate and RNA was extracted in Trizol (Invitrogen). Library preparation and sequencing was carried out by the NYU Genome Technology Center. RNA-Seq library preps were made using the Illumina TruSeq RNA sample Prep Kit v2 (Cat #RS-122-2002), using 500 ng of total RNA as input, amplified by 12 cycles of PCR, and run on an Illumina 2500 (v4 chemistry), as single read 50. For each RNA-seq sample, sequence quality was assessed with FastQC (http://www.bioinformatics.babraham.ac.uk/projects/fastqc) and sequencing adapters were removed with Trimmomatic [98]. Cleaned reads were aligned to the *Drosophila* reference genome (dm3) with Tophat2 v2.1.1 1. The Picard CollectRnaSeqMetrics program (https://broadinstitute.github.io/picard/picard-metric-definitions.html#RnaSeqMetrics) was used to generate QC metrics including ribosomal RNA content, median per-gene coverage, bases aligned to intergenic regions, 5’/3’ biases, and the distribution of the bases within exons, UTRs, and introns. Per sample gene expression profiles were computed using Cufflinks v2.2.1 1 and the RefSeq genome annotation for the *Drosophila* reference genome dm3 [99]. For multi-sample comparison, Principal Component Analysis and hierarchical clustering were used to verify that the expression profiles of the sequenced samples clustered as expected by sample tissue and genotype. Differential gene expression was computed for various contrasts between genotypes with the Cufflinks protocol [100] with default thresholds. Reads on the *gl* gene were visualized with Integrative Genomics Viewer (IGV, Broad Institute). Genes were considered significantly changed and included in the heat map if the log_2_ fold change for either wing or brain misexpression was >1, p<0.01, average cpm for that misexpression condition was >1, and standard deviation/mean of the three replicates was <0.5. Intron sizes were obtained from Flybase and compared for targets predicted with high confidence by [40] that were induced (log fold change >1 in either or both tissues) or not induced (log fold change <0.1 in both tissues). Homer was used to identify motifs enriched in the genes included in the heat map compared to predicted targets that were not induced in either tissue, using a region that extended 2 kb upstream and downstream of each gene. RNA-Seq data have been submitted to NCBI GEO (reference number GSE99303).

## Supporting information

S1 Fig Gl can induce Chp in the embryonic CNS. All panels show late stage embryos stained with anti-Chp (A’, B’, C’, D’, E’, magenta in A-E). (A) is stained with anti-Elav (green). *UAS-mCD8GFP* (green) and *UAS-gl* are not expressed (A) or expressed with *dpn-GAL4* (B), *elav-GAL4* (C), *repo-GAL4* (D) or *ato-GAL4* (E). Expressing Gl in either neuroblasts or neurons induces ectopic Chp in the CNS, but expressing Gl in glial cells or in the peripheral nervous system has no effect. Scale bars: 50μm.
(TIF)

S2 Fig Gl expressed in neuroblasts is maintained in their progeny. All panels show third instar larval brains stained with anti-Gl (A’, B’, C’, red in A-C), anti-Chp (D’, red in D) and anti-Elav (blue). mCD8GFP (green) and Gl are driven by *insc-GAL4* in (B-D), with *tub-GAL80*^*ts*^ to bypass early lethality. Animals were reared at 18°C for three days and then shifted to 29°C. Cells that express Gl or Chp but not GFP are indicated by arrowheads in (C, D). These cells express Elav and are therefore likely to be differentiated neuronal progeny of the Gl-expressing neuroblasts. Scale bars: 100μm in (A,B); 50μm in (C, D).
(TIF)

S3 Fig The size of the region searched for Gl binding sites is inversely correlated with the probability that a predicted Gl target is induced. (A) Gl target genes predicted by (Potier et al., 2014) based on the presence of Gl binding motifs in a region consisting of 5 kb upstream and the first intron are binned according to the size of their first intron and plotted as induced by Gl in one or more tissues (black) or induced in neither (grey). (B) A plot of the log fold change in Gl-expressing wing discs (non-patterned bars) or brains (patterned bars) for the 13 predicted targets that were induced by Gl, divided according to the size of their first introns (intron size 0–500 (red), 501–1000 (green), 1001–5000 (yellow), >5000 (blue)). Interestingly, 5 of these genes are highly enriched in mature photoreceptors (*HisCl1*, *Ekar*, *inaC*, *chp*, *nrm*) and 6 in cone cells (*CG9150*, *cd*, *CG5653*, *sls*, *CG11498*, *Cad88C*).
(TIF)

S4 Fig Somatic CRISPR effectively mutates *gl*. (A) Schematic showing the positions in the *gl* gene targeted by the two sgRNAs. Noncoding regions are shown in gray and the zinc fingers in white. (B) wild-type control and (C, D) *ey3*.*5-FLP*, *Act>CD2>GAL4; gl sgRNAs; UAS-Cas9P2/gl*^*60j*^. (B, C) show larval eye discs stained with anti-Gl (B’, C’, magenta in B, C) and anti-Elav (green), and (D) shows an adult eye. Expressing Cas9 throughout the eye disc in a *gl* heterozygote results in mosaic loss of Gl by the third instar and a moderate *gl* mutant phenotype in the adult. (E) wild type; (F, G) *elav-GAL4/gl sgRNAs; UAS-Cas9P2/gl*^*60j*^. (E, F) show 42h APF pupal retinas stained with anti-Gl (E’, F’, magenta in E, F), anti-Elav and anti-Ecad (both in green) and (G) shows an adult eye. Insets are enlargements of single boxed ommatidia. Gl staining is reduced in photoreceptors but still present in pigment cells. Expressing Cas9 in photoreceptors results in a weaker, mosaic *gl* mutant phenotype (G). (H-J) show third instar larval eye discs stained with anti-Pax2 (J, green in H, I) to mark cone cells and anti-Gl (H’, I’, magenta in H, I). (H) wild type; (I) *spa-GAL4; gl sgRNAs; UAS-Cas9P2/gl*^*60j*^; (J) *gl*^*60j*^. Gl is lost from some Pax2^+^ cone cells (circled by yellow dashed line in I). (K) *54-GAL4*, *UAS-lacZ*; (L) *54-GAL4*, *UAS-lacZ /gl sgRNAs; UAS-Cas9P2/gl*^*60j*^ 42h APF pupal retinas stained with anti-Gl (K’, L’, red in K, L) and anti-β-galactosidase (green). Gl is lost from some pigment cells. Insets show enlargements of single boxed ommatidia. Scale bars: 50μm in (B’,C’); 10μm in (E’,F’,K’,L’); 20μm in (H’,I’); 30μm in (J).
(TIF)

S5 Fig Quantification of defects in retinas lacking *gl* in cone cells or pigment cells. (A-J) show individual ommatidia from 42h APF pupal retinas, stained with anti-Ecad. Wild-type (A, F), *spa-GAL4; gl sgRNAs; UAS-Cas9P2/gl*^*60j*^ (B-E) and *54-GAL4*, *UAS-lacZ /gl sgRNAs; UAS-Cas9P2/gl*^*60j*^ (G-J). Loss of Gl in cone cells or pigment cells results in ommatidial patterning defects. Scale bars: 5μm. (K) Quantification of Elav^+^ cells per ommatidium observed in cell-specific CRISPR experiments compared to *gl* sgRNAs; UAS-Cas9P2, *gl*^*60j*^*/+* control. Loss of Gl in non-neuronal cells does not affect photoreceptor numbers.
(TIF)

S1 Table Lists of the genes shown in the heat map in Fig 4A and the Euler diagram in Fig 4B. Separate sheets show the genes induced when *gl* was misexpressed with *insc-GAL4* in *gl* mutant larval brains compared to *gl* mutant brains, genes induced when *gl* was misexpressed in clones made with *Ubx-FLP* in wing discs compared to wild type wing discs, genes more highly expressed in wild type third instar eye discs than wing discs, and genes more highly expressed in wild type third instar eye discs than brains, all using the same cutoffs (fold change>2, p<0.01, average counts in eye discs >1 and standard deviation/mean of eye disc samples<0.5). The final sheet shows predicted direct targets of Gl according to [40]. Columns show CPM in each of the three samples of each tissue and the log_2_ fold changes and p values for the indicated comparisons.
(XLSX)

S2 Table Quantification of patterning defects caused by somatic CRISPR. Numbers of the indicated defects in photoreceptor number, cone cell, pigment cell or bristle cell number or arrangement observed in cell type-specific *gl* mutants and controls.
(XLSX)
