# Supplementary figures and images for: Glass promotes the differentiation of neuronal and non-neuronal cell types in the Drosophila eye
Source: PLoS Genet. 2018 Jan 11;14(1):e1007173. doi: 10.1371/journal.pgen.1007173 (PMC5783423; doi:10.1371/journal.pgen.1007173)

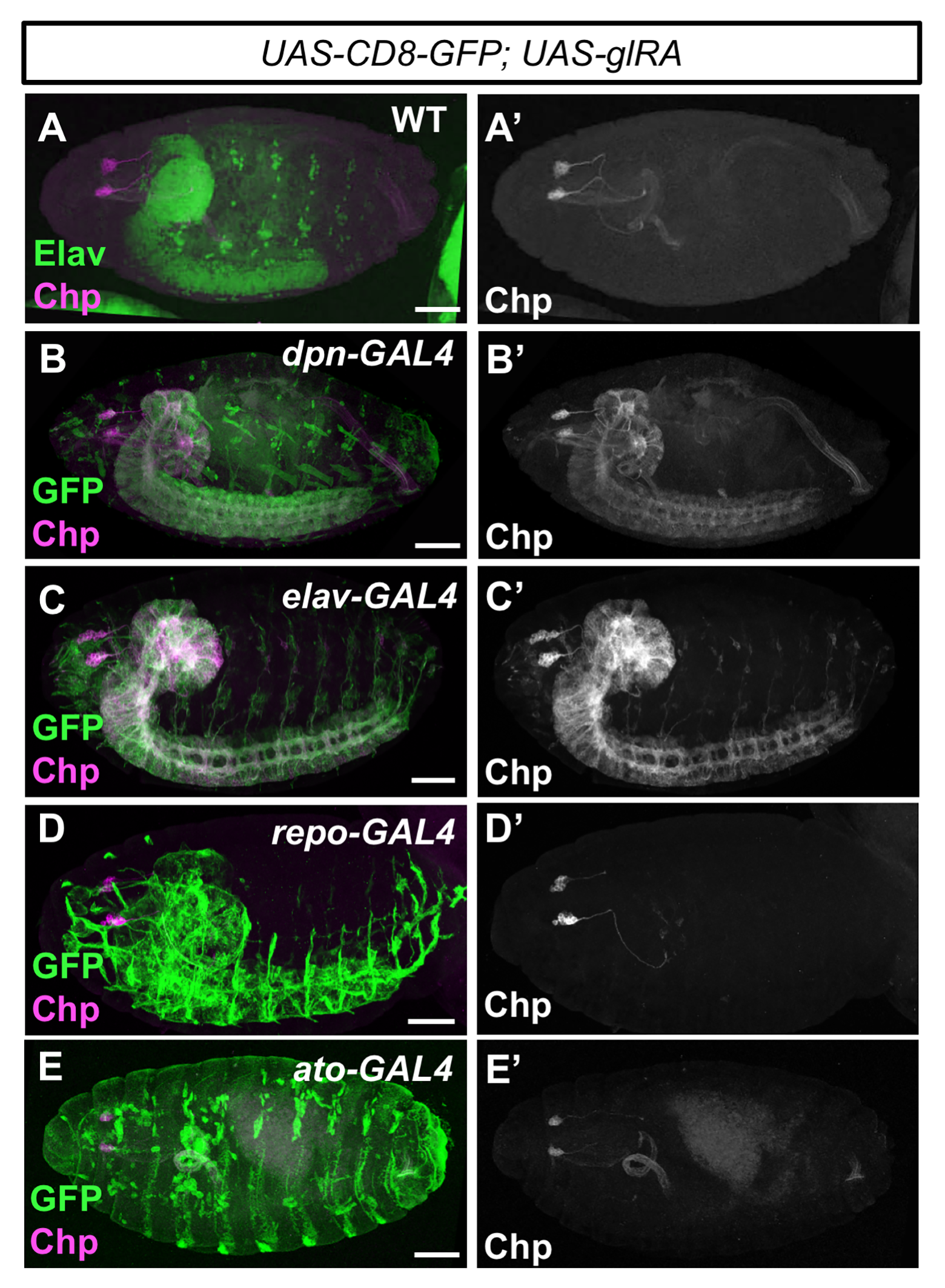

Supplement: S1 Fig — All panels show late stage embryos stained with anti-Chp (A’, B’, C’, D’, E’, magenta in A-E). (A) is stained with anti-Elav (green). UAS-mCD8GFP (green) and UAS-gl are not expressed (A) or expressed with dpn-GAL4 (B), elav-GAL4 (C), repo-GAL4 (D) or ato-GAL4 (E). Expressing Gl in either neuroblasts or neurons induces ectopic Chp in the CNS, but expressing Gl in glial cells or in the peripheral nervous system has no effect. Scale bars: 50μm. (TIF) [file pgen.1007173.s001.tif]

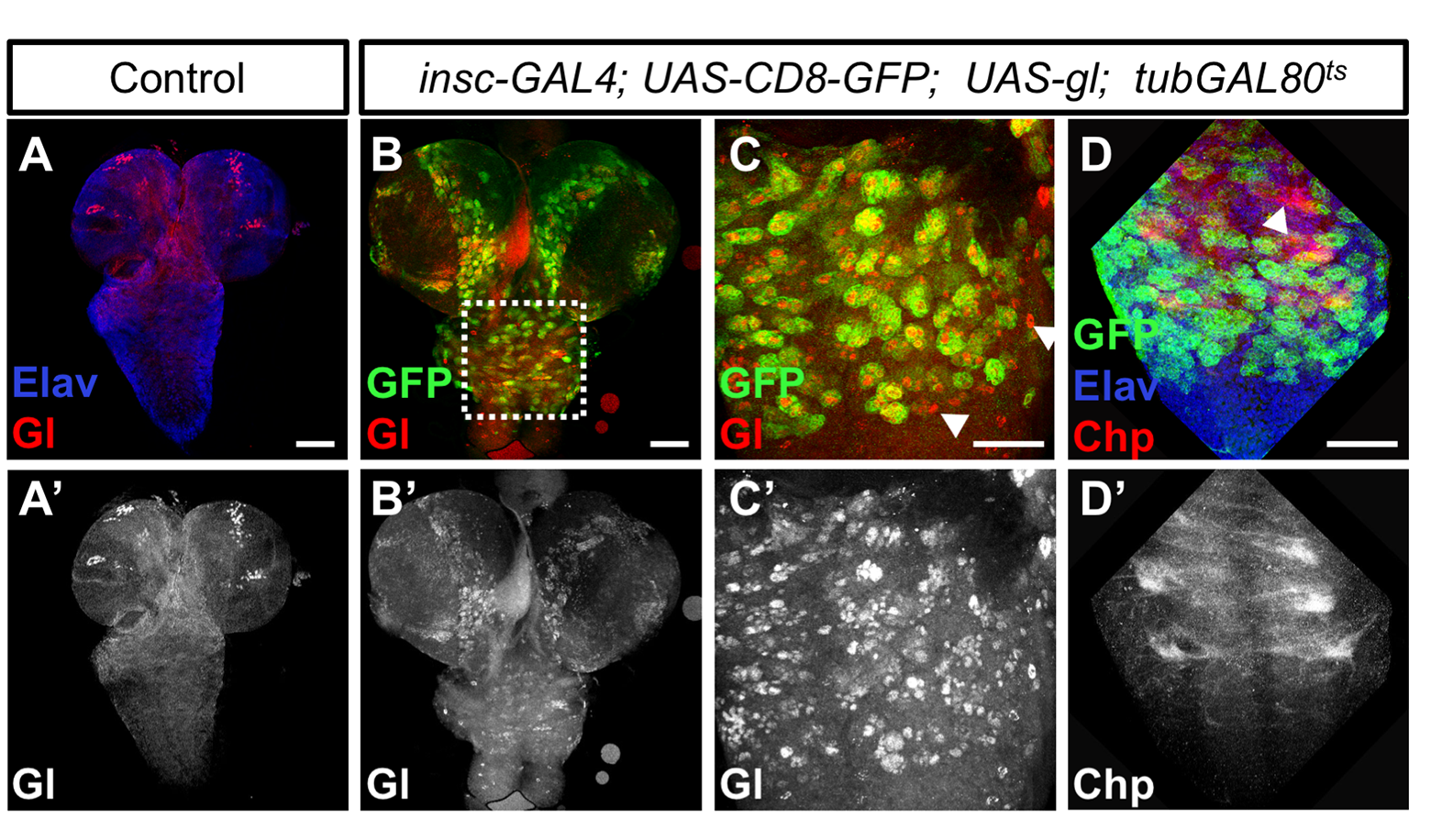

Supplement: S2 Fig — All panels show third instar larval brains stained with anti-Gl (A’, B’, C’, red in A-C), anti-Chp (D’, red in D) and anti-Elav (blue). mCD8GFP (green) and Gl are driven by insc-GAL4 in (B-D), with tub-GAL80ts to bypass early lethality. Animals were reared at 18°C for three days and then shifted to 29°C. Cells that express Gl or Chp but not GFP are indicated by arrowheads in (C, D). These cells express Elav and are therefore likely to be differentiated neuronal progeny of the Gl-expressing neuroblasts. Scale bars: 100μm in (A,B); 50μm in (C, D). (TIF) [file pgen.1007173.s002.tif]

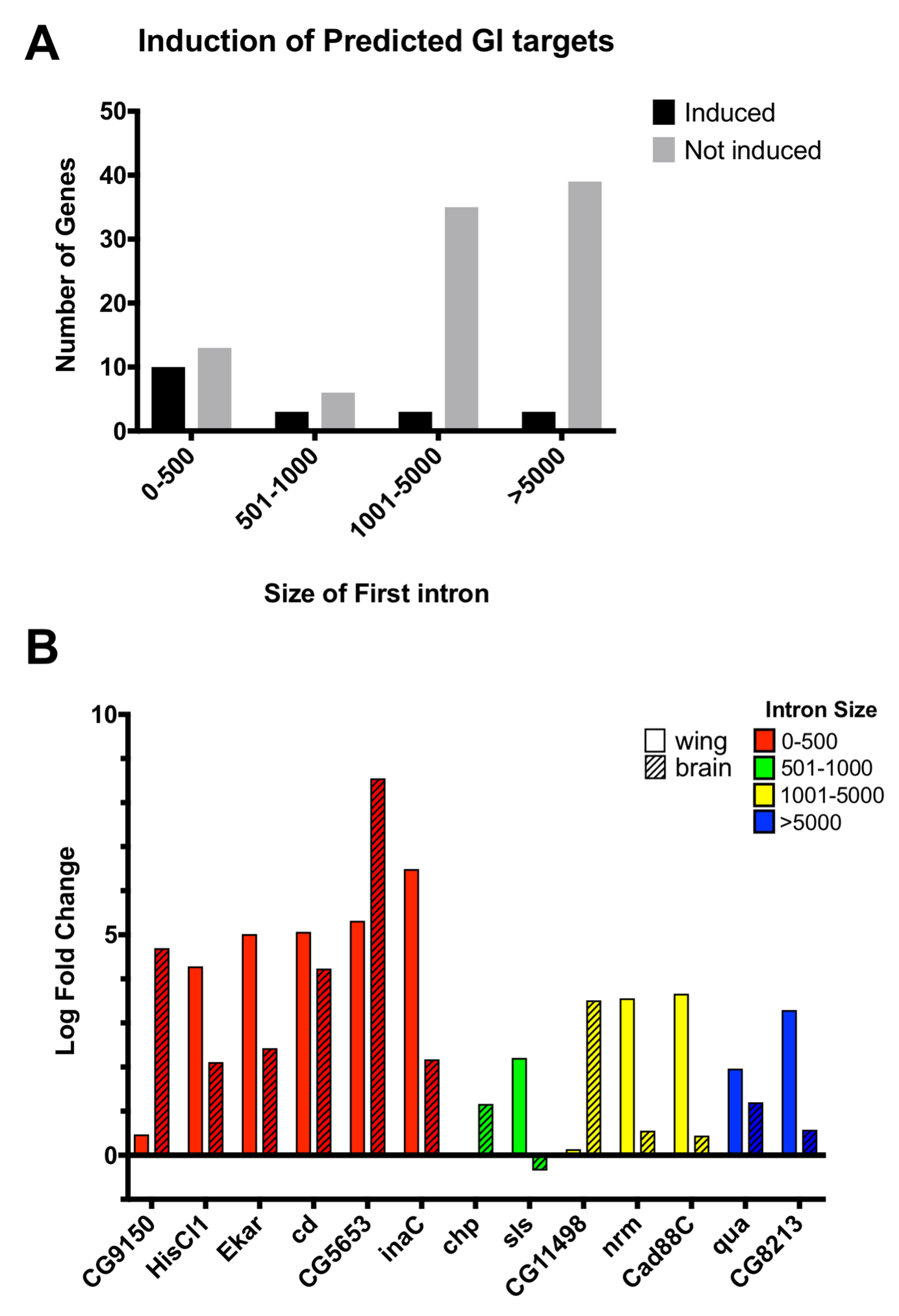

Supplement: S3 Fig — (A) Gl target genes predicted by (Potier et al., 2014) based on the presence of Gl binding motifs in a region consisting of 5 kb upstream and the first intron are binned according to the size of their first intron and plotted as induced by Gl in one or more tissues (black) or induced in neither (grey). (B) A plot of the log fold change in Gl-expressing wing discs (non-patterned bars) or brains (patterned bars) for the 13 predicted targets that were induced by Gl, divided according to the size of their first introns (intron size 0–500 (red), 501–1000 (green), 1001–5000 (yellow), >5000 (blue)). Interestingly, 5 of these genes are highly enriched in mature photoreceptors (HisCl1, Ekar, inaC, chp, nrm) and 6 in cone cells (CG9150, cd, CG5653, sls, CG11498, Cad88C). (TIF) [file pgen.1007173.s003.tif]

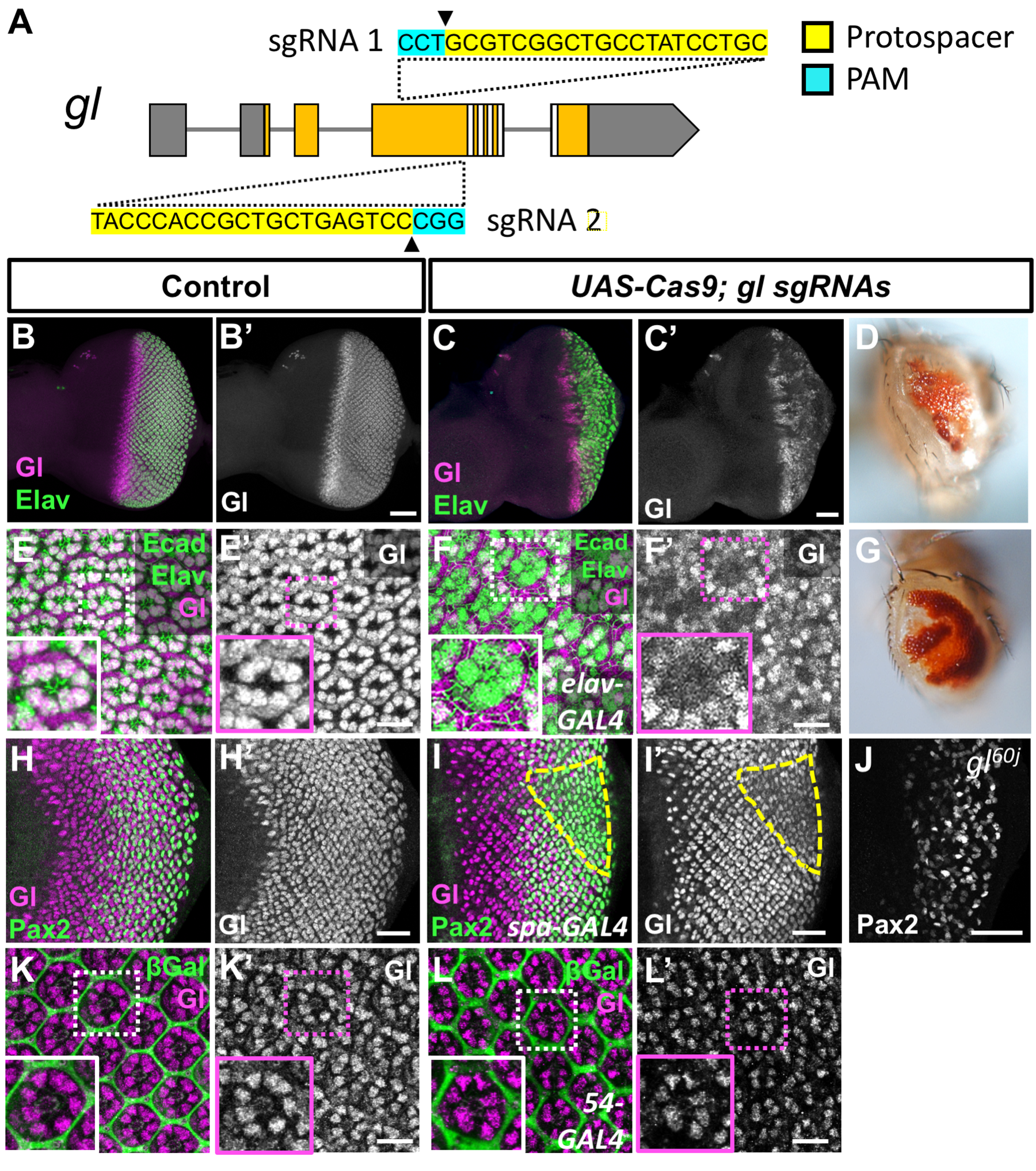

Supplement: S4 Fig — (A) Schematic showing the positions in the gl gene targeted by the two sgRNAs. Noncoding regions are shown in gray and the zinc fingers in white. (B) wild-type control and (C, D) ey3.5-FLP, Act>CD2>GAL4; gl sgRNAs; UAS-Cas9P2/gl60j. (B, C) show larval eye discs stained with anti-Gl (B’, C’, magenta in B, C) and anti-Elav (green), and (D) shows an adult eye. Expressing Cas9 throughout the eye disc in a gl heterozygote results in mosaic loss of Gl by the third instar and a moderate gl mutant phenotype in the adult. (E) wild type; (F, G) elav-GAL4/gl sgRNAs; UAS-Cas9P2/gl60j. (E, F) show 42h APF pupal retinas stained with anti-Gl (E’, F’, magenta in E, F), anti-Elav and anti-Ecad (both in green) and (G) shows an adult eye. Insets are enlargements of single boxed ommatidia. Gl staining is reduced in photoreceptors but still present in pigment cells. Expressing Cas9 in photoreceptors results in a weaker, mosaic gl mutant phenotype (G). (H-J) show third instar larval eye discs stained with anti-Pax2 (J, green in H, I) to mark cone cells and anti-Gl (H’, I’, magenta in H, I). (H) wild type; (I) spa-GAL4; gl sgRNAs; UAS-Cas9P2/gl60j; (J) gl60j. Gl is lost from some Pax2+ cone cells (circled by yellow dashed line in I). (K) 54-GAL4, UAS-lacZ; (L) 54-GAL4, UAS-lacZ /gl sgRNAs; UAS-Cas9P2/gl60j 42h APF pupal retinas stained with anti-Gl (K’, L’, red in K, L) and anti-β-galactosidase (green). Gl is lost from some pigment cells. Insets show enlargements of single boxed ommatidia. Scale bars: 50μm in (B’,C’); 10μm in (E’,F’,K’,L’); 20μm in (H’,I’); 30μm in (J). (TIF) [file pgen.1007173.s004.tif]

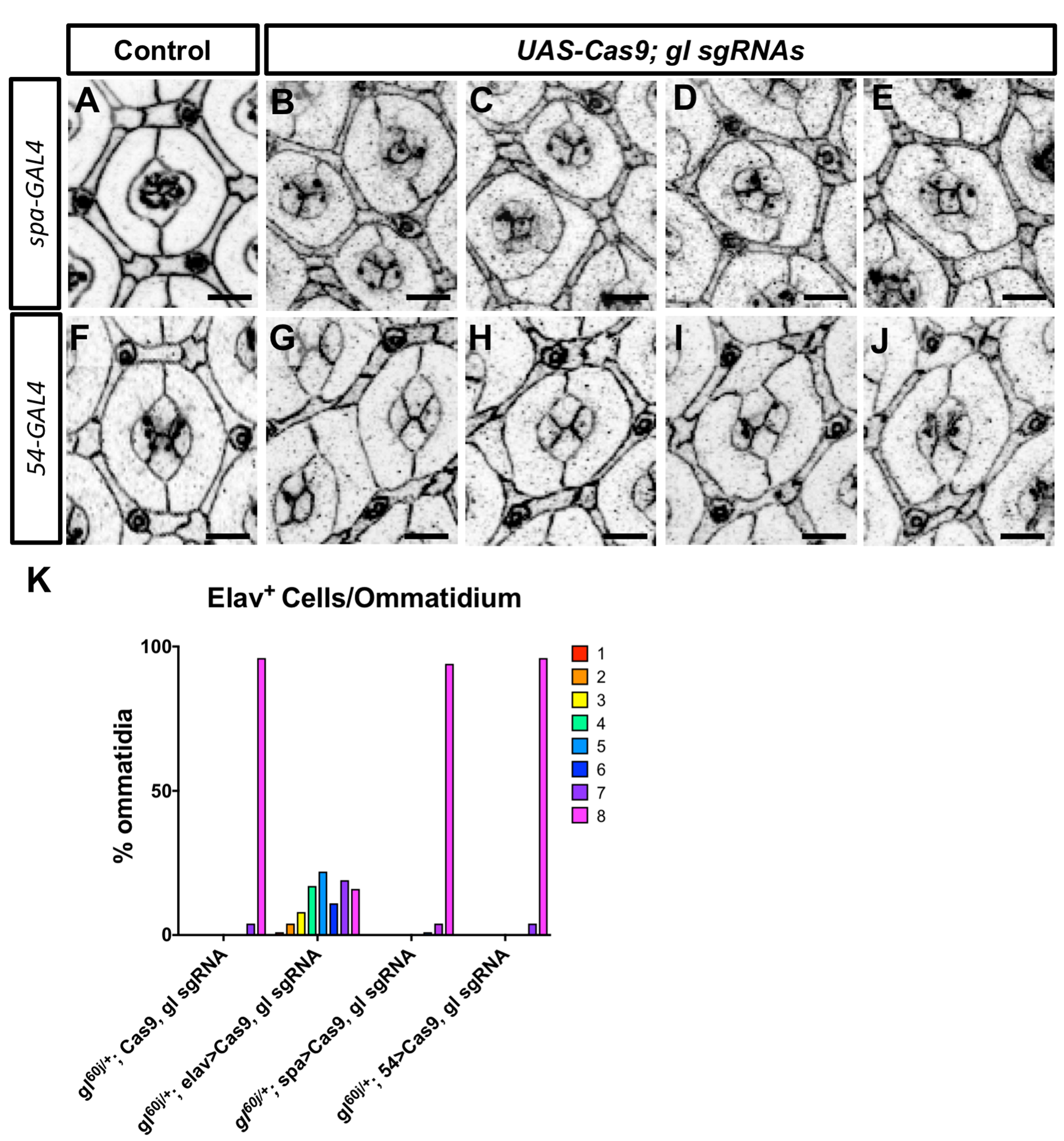

Supplement: S5 Fig — (A-J) show individual ommatidia from 42h APF pupal retinas, stained with anti-Ecad. Wild-type (A, F), spa-GAL4; gl sgRNAs; UAS-Cas9P2/gl60j (B-E) and 54-GAL4, UAS-lacZ /gl sgRNAs; UAS-Cas9P2/gl60j (G-J). Loss of Gl in cone cells or pigment cells results in ommatidial patterning defects. Scale bars: 5μm. (K) Quantification of Elav+ cells per ommatidium observed in cell-specific CRISPR experiments compared to gl sgRNAs; UAS-Cas9P2, gl60j/+ control. Loss of Gl in non-neuronal cells does not affect photoreceptor numbers. (TIF) [file pgen.1007173.s005.tif]
